# Synthesis, Radiosynthesis and Biological Evaluation of Buprenorphine‐Derived Phenylazocarboxamides as Novel μ‐Opioid Receptor Ligands

**DOI:** 10.1002/cmdc.202000180

**Published:** 2020-06-02

**Authors:** Jasmin Krüll, Stefanie K. Fehler, Laura Hofmann, Natascha Nebel, Simone Maschauer, Olaf Prante, Peter Gmeiner, Harald Lanig, Harald Hübner, Markus R. Heinrich

**Affiliations:** ^1^ Department of Chemistry and Pharmacy Pharmaceutical Chemistry Friedrich-Alexander-Universität Erlangen-Nürnberg Nikolaus-Fiebiger-Str. 10 91058 Erlangen Germany; ^2^ Department of Nuclear Medicine Molecular Imaging and Radiochemistry Friedrich-Alexander-Universität Erlangen-Nürnberg Schwabachanlage 12 91054 Erlangen Germany; ^3^ Central Institute for Scientific Computing (ZISC) Friedrich-Alexander-Universität Erlangen-Nürnberg Martensstr. 5a 91058 Erlangen Germany

**Keywords:** azo compounds, bioisosters, buprenorphine, opioid receptors, radiosynthesis

## Abstract

Targeted structural modifications have led to a novel type of buprenorphine‐derived opioid receptor ligand displaying an improved selectivity profile for the μ‐OR subtype. On this basis, it is shown that phenylazocarboxamides may serve as useful bioisosteric replacements for the widely occurring cinnamide units, without loss of OR binding affinity or subtype selectivity. This study further includes functional experiments pointing to weak partial agonist properties of the novel μ‐OR ligands, as well as docking and metabolism experiments. Finally, the unique bifunctional character of phenylazocarboxylates, herein serving as precursors for the azocarboxamide subunit, was exploited to demonstrate the accessibility of an ^18^F‐fluorinated analogue.

## Graphical abstract

**Targeted replacement** of a cinnamide for a phenylazocarboxamide moiety led to a novel type of buprenorphine‐derived opioid receptor ligand that display selectivity for the μ‐subtype. Besides good metabolic stability and no detectable cytotoxicity, the phenylazocarboxamide unit enabled straightforward access to an ^18^F‐fluorinated analogue, thus making this substructure a promising building block for future research in medicinal chemistry.

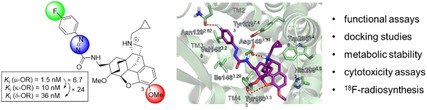

## Introduction

**Figure 1:**
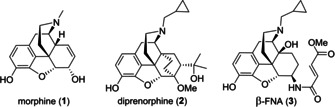
Selected examples of morphinan‐derived opioid receptor ligands.

Opioid receptors (OR) play an important role in medicinal chemistry^1^, ^2^, ^3^, ^4^, ^5^ and continuous efforts are made to develop new drug candidates^1^, ^6^, ^7^, ^8^ as well as ligands designed for investigations in the chemical biology of these receptors. Among the major subtypes μ,^9^ κ,^10^ δ,^11^ and the nociceptin receptor^12^ the selective addressing of the μ subtype is of interest for a number of applications.^13^, ^14^, ^15^, ^16^, ^17^, ^18^ For example, the selective μ‐OR agonist PZM21 has been proposed for pain treatment with reduced side effects.^19^ PZM21 is not only subtype selective, but also displays biased signaling with minimal β‐arrestin recruitment.^20^, ^21^ Further analogues of PZM21 were recently reported by Shi and co‐workers.^22^ The partial μ‐OR agonist NAP, which contains a morphinan scaffold, also shows biased signal transduction.^23^ NAP was developed for the treatment of opioid‐induced constipations. A first‐in‐human clinical trial was already performed applying the biased μ‐OR agonist TRV130.^24^, ^25^ Although pain management with reduced side effects such as respiratory depression was observed within this clinical trial, the value of TRV130 has proven to be controversial.^26^ Upon optimization of a piperidine benzimidazolone scaffold, promising μ‐OR agonists with exceptionally high bias factors have recently been discovered by Bohn and Bannister.^27^ Regarding the recently developed OR ligands from a structural point of view, non‐morphinan‐derived^19^, ^22^, ^24^, ^27^ as well as morphinan‐derived^23^, ^28^, ^29^, ^30^, ^31^ scaffolds have been used. Among the latter ones, scaffolds based on morphine (**1**),^32^ diprenorphine (**2**)^33^ and β‐funaltrexamine (*β*‐FNA) (**3**)^34^, ^35^ still represent valuable starting points in the development of opioid receptor ligands (Figure 1).^36^

With regard to subtype selectivity, the particular importance of the side chain on the diprenorphine scaffold can be derived from the binding data summarized in Table 1.^33^, ^34^, ^37^, ^38^

**Table 1:** Binding affinities of known ligands derived from diprenorphine (**2**).

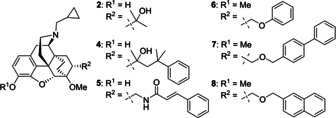				
compound	R^1^=	*K*_i_ [nM]		
		μ	κ	δ
**2** 33	H	0.14	2.0	0.73
**4** 37	H	2.19±0.65	4.15±1.3	3.66±0.92
**5** 34	H	0.7±0.25	2.6±0.0	0.7±0.05
**6** 38	Me	14.4	0.15	89.3
**7** 38	Me	1.91	25.8	1753
**8** 38	Me	1.87	0.74	–

When aiming at μ subtype selectivity, the 3‐hydroxy substituted ligands diprenorphine (**2**), **4** and **5** (R^1^=H) do not appear as preferred lead structures since these compounds show limited subtype discrimination. Methylation of the 3‐hydroxy group (R^1^=Me), however, changes the influence of the substituent R^2^. As demonstrated by the diprenorphine derivatives **6–8**,^38^ and in particular by the biphenyl derivative **7**, a suitably chosen side chain R^2^ can now lead to preferred binding to the μ subtype.

**Scheme 1:**
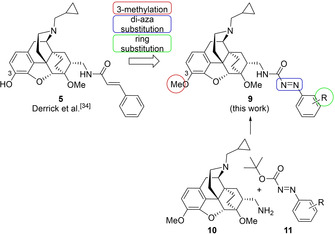
Structural modifications of cinnamide ligand **5** and synthetic approach to azocarboxamides **9** from amine **10** and *tert*‐butyl phenylazocarboxylates **11**.

For this study, we thought to combine the high binding affinity of the cinnamide **5** 34 towards the μ‐OR (*K*_i_=0.7 nM, Table 1) with methylation at the 3‐hydroxy group to shift the selectivity towards the μ subtype (Scheme 1). Moreover, the cinnamide substructure should be replaced by a phenylazocarboxamide so that ligands of the general structure **9** would be obtained.

Besides our general interest in the question whether phenylazocarboxamides can serve as bioisosteric replacements for cinnamide substructures,^39^, ^40^ the successful preparation of the target compounds **9** from amine **10** and azo esters **11** would further enable a straightforward access to the corresponding ^18^F‐fluorinated analogue (R=4‐^18^F; Scheme 1). This is due to the good availability of the ^18^F‐labeled *tert*‐butyl phenylazocarboxylate **11** (R=4‐^18^F),^41^, ^42^, ^43^ whereat the corresponding ^18^F‐labeled azocarboxamide **9** (R=4‐^18^F) could then serve as a μ‐OR radioligand for *in vivo* PET imaging studies.^42^, ^43^, ^44^, ^45^, ^46^ For all OR ligands of the general structure **9**, non‐radiolabeled as well as radiolabeled derivatives, we envisaged the coupling of the amine **10** with the corresponding *tert*‐butyl phenylazocarboxylate **11** as final synthetic step.^41^ In addition to chemical and radiochemical syntheses, computational docking studies were carried out to give insights into the factors responsible for binding affinity and subtype selectivity. Besides the potential cytotoxicity, we further investigated the metabolic stability of the novel ligands using an in vitro rat microsomal stability assay.

## Results and Discussion

**Scheme 2:**
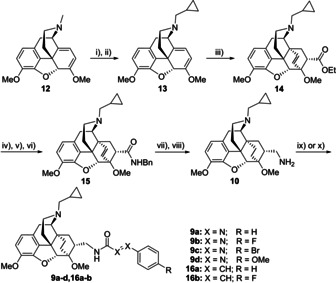
Synthesis of azocarboxamide ligands **9 a**–**d** and reference compounds **16 a**,**b**. i) *m*CPBA, HCl, ferroceneacetic acid (12 mol%), CHCl_3_/*i*PrOH (3 : 1), −5 °C to 50 °C, 28 h, 80 %; ii) (bromomethyl)cyclopropane, NaHCO_3_, DMF, 90 °C, 20 h, 65 %; iii) ethyl acrylate, 100 °C, 15 h, 78 %; iv) HCl (6 m), 100 °C, quant.; v) oxalyl chloride, DMF (cat.), CHCl_3_, 0 °C to RT, 23 h; vi) benzylamine, NEt_3_, CHCl_3_, 0 °C to RT 22 h, 79 % (over two steps); vii) LiAlH_4_, THF, 70 °C, 20 h, 51 %; viii) Pd/C (10 %), ammonium formate, EtOH, 90 °C, 1.5 h, 81 %; ix) *tert*‐butyl phenyl‐azocarboxylate **11 a**–**d**, K_2_CO_3_ or NEt_3_, EtOH, RT, 3–120 h, 42–87 %; x) cinnamic acid chloride **17 a**,**b**, NaHCO_3_, CH_2_Cl_2_, RT, 28 h, 25–63 %.

The synthetic route to the azocarboxamide and cinnamide ligands **9 a**–**d** and **16 a**–**b** evaluated in this work is depicted in Scheme 2. For some reactions, the conditions are based on those previously established by Kok et al.,^47^ and for other steps, the reaction conditions were derived from the work by Derrick et al.^34^

The ligand synthesis started with the demethylation of thebaine (**12**) using ferroceneacetic acid, *m*CPBA and hydrochloric acid to yield a secondary amine, followed by an N‐alkylation of this amine with (bromomethyl)cyclopropane in the presence of NaHCO_3_ to obtain the alkylated compound **13**. Afterwards, ethyl acrylate was used as dienophile for a hetero Diels‐Alder reaction with diene **13** to give ester **14**. The ester **14** was hydrolyzed under acidic conditions, the resulting carboxylic acid was activated with oxalyl chloride, and the acid chloride was trapped with benzylamine to furnish the amide **15**. The primary amine **10** was prepared from **15** by reduction of the amide moiety by LiAlH_4_ and subsequent hydrogenation of the double bond which was accompanied by cleavage of the benzyl protecting group. At this point, it is worth to note that the carbon‐carbon double bond was already partially reduced under the LiAlH_4_ conditions of step vii). For step viii), the procedure by Derrick^34^ recommended the use of H_2_ and Pd/C at 45 °C under a H_2_ pressure of 30 psi (ca. 2.1 bar) to give a yield of 76 %. We initially conducted the hydrogenation under atmospheric pressure conditions (H_2_ balloon), leaving all other reagents and conditions unchanged, and obtained the primary amine **10** in 68 % yield. Even after extended reaction times, only partial conversion of the starting material had taken place, and after the addition of a larger excess of acidic acid, we detected the opening of the cyclopropyl ring *via* LC/MS analysis. Thus, the conditions were changed to Pd/C and ammonium formate at a reaction temperature of 90 °C. Under these conditions, the primary amine **10** was obtained in 81 % yield after 2.5 h, and the desired product could now be easily purified by column chromatography. The synthesis of the azocarboxamide ligands **9 a**–**d** was accomplished by a coupling of the primary amine **10** to the corresponding *tert*‐butyl phenylazocarboxylates **11 a**–**d** in the presence of sodium bicarbonate or triethylamine. Among the four different azocarboxylic esters used in step ix), slightly modified conditions were required for the *tert*‐butyl 4‐fluorophenylazocarboxylate (**11 b**), as the long reaction time of three days led to a partial substitution of the 4‐fluoro substituent by an ethoxy group originating from the solvent ethanol. Since ethanol turned out to be the best choice compared to other solvents, the synthesis of the fluorinated ligand **9 b** was conducted at increased concentrations and with a larger excess of the azocarboxylate **11 b**. In this way, the reaction time could be reduced from three days to 2.5 h and the side reaction to the aryl ethyl ether could be suppressed. In step x), the cinnamide ligands **16 a** and **16 b** were prepared from amine **10** and the related cinnamic acid chlorides **17 a,b** in the presence of sodium bicarbonate.

The binding affinities of the azocarboxamides **9 a**‐**d** and the cinnamides **16 a,b** to the μ‐, κ‐ and δ‐OR subtypes were determined by radioligand competition binding assays using [^3^H]diprenorphine and membrane preparations derived from HEK293T cells transiently expressing the related receptor subtypes. Displacement curves resulted in *K*_i_ values for **9 a**–**d, 16 a,b** and the reference β‐FNA (**3**) (Table 2).

**Table 2:** Binding affinities of compounds **3**, **5**, **9 a**–**d** and **16 a,b** towards the μ‐, κ‐ and δ‐OR subtypes.

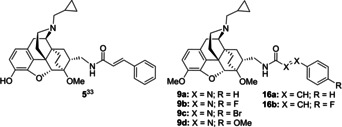					
Compound	*K*_i_ (nM±SEM)^[a]^			Selectivity^[b]^	
	μ	κ	δ	μ/κ	μ/δ
β‐FNA (**3**)^*c*^	0.4±0.05	0.9±0.05	7.7±2.4	2	20
**3**	1.5±0.21	10±2.3	26±7.4	6.7	17
**5** ^*c*^	0.7±0.25	2.6±0.0	0.7±0.05	4	1
**9 a**	2.3±0.26	19±1.9	30±2.8	8.3	13
**9 b**	1.5±0.45	10±2.2	36±6.5	6.7	24
**9 c**	1.3±0.30	10±2.6	28±3.0	7.7	22
**9 d**	4.0±0.39	11±1.4	63±9.9	2.8	16
**16 a**	4.9±1.5	18±5.7	54±9.1	3.7	11
**16 b**	3.3±0.8	25±5.1	58±11	7.6	18

[a] Binding affinities are displayed as mean *K*_i_ values ±SEM derived from three to 11 individual experiments each done in triplicate. [b] Subtype selectivity for μ‐OR expressed as ratio of *K*_i_ values. [c] Binding affinities as reported by Derrick et al.^34^

In the previous biological evaluation of cinnamide **5**,^34^ which served as a starting point for the design of our azocarboxamide ligands **9 a**–**d**, β‐FNA (**3**) was used as a reference compound (Table 2). Under our assay conditions, the *K*_i_ values determined for β‐FNA (**3**) were all increased by factors in the range of three to 11 (×4 for the μ‐OR, ×11 for the κ‐OR and ×3.5 for the δ‐OR) thereby suggesting that our assay is less sensitive for all three OR subtypes. At this point it is important to remember that β‐FNA (**3**) is known to be an irreversible ligand at the μ‐OR, so that the equilibrium binding affinity to this particular subtype can be highly sensitive to deviations in the assay conditions. As the *K*_i_ values for *all* three OR subtypes are however altered by comparable factors, and binding of β‐FNA (**3**) to the κ‐OR and the δ‐OR subtype is reversible, a certain comparison of our data with that available for **5** 34 appears possible.

Taking into account the above mentioned sensitivity factors (×4 for the μ‐OR, ×11 for the κ‐OR and ×3.5 for the δ‐OR), one can assume that cinnamide **5** and our ligands **9 a**–**d** and **16 a,b** show comparable binding affinities at the μ‐OR and κ‐OR subtypes. At the δ‐OR, in contrast, the binding of **5** is significantly stronger. Methylation at the 3‐hydroxy group, which constitutes the general structural difference between the reference compound **5** and all of our ligands **9 a**–**d** and **16 a,b**, thus leads to an improved selectivity profile for the μ towards the δ subtype, but not for μ compared to κ. This analysis is also reflected by the selectivity ratios κ/μ and δ/μ reported in Table 2.

Among our ligands **9 a**–**d** and **16 a,b** neither di‐aza substitution nor ring substitution on the azocarboxamide (c.f. Scheme 1) led to strong changes in binding affinity at any of the three OR subtypes, thereby indicating that these structural modifications are tolerated and that no major ligand‐receptor interactions are changed. Upon comparison of the newly prepared azocarboxamides **9 a**–**d** and cinnamides **16 a,b**, the 4‐fluoro and the 4‐bromo azocarboxamide derivatives **9 b** and **9 c** showed the most favorable biological profiles with regard to the μ‐OR. The highest binding affinities were determined for **9 b** and **9 c** and the subtype selectivity ratios κ/μ and δ/μ for these ligands were also among the best values.

Because a covalent binding mode to the μ‐OR was originally considered for the reference compound **5**,^34^ radioligand depletion assays were performed for the novel azocarboxamide **9 b** and its corresponding cinnamide **16 b**. Through these assays it turned out that a covalent binding to the μ‐OR is neither likely for **9 b**, nor for **16 b**, whereas **16 b** is more closely related to reference compound **5** due to the common cinnamide substructure.

**Figure 2:**
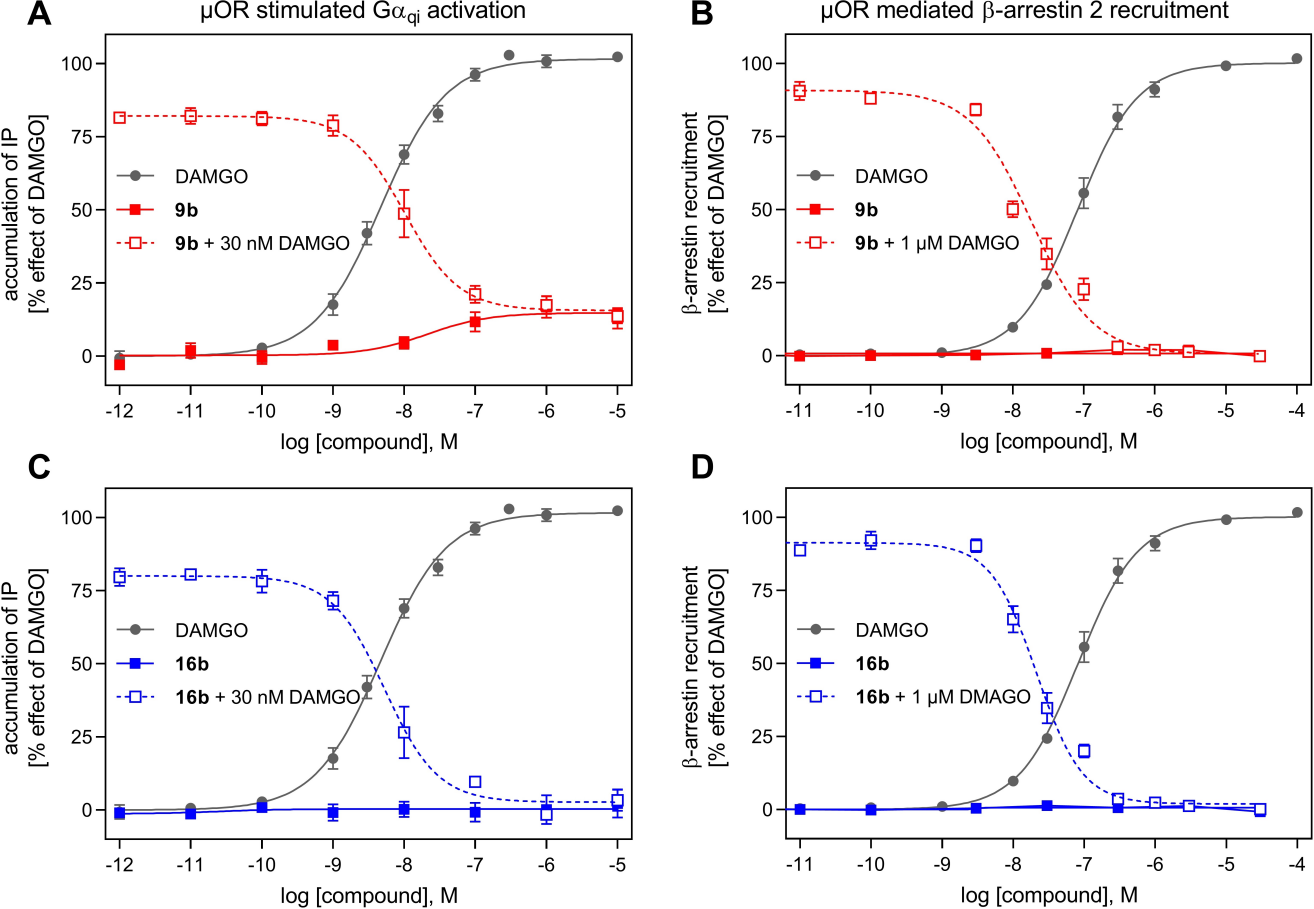
Functional investigation of the selected test compounds applying an IP accumulation assay (IP‐One^®^) for testing G‐protein signaling and an arrestin‐2 recruitment assay (Path Hunter^®^). A, C) G‐protein signaling was determined in HEK‐293T cells transiently co‐transfected with μ‐OR and the hybrid G protein Gα_qi_ (Gα_q_ protein with the last five amino acids at the C terminus replaced by the corresponding sequence of Gα_i_). Whereas **9 b** (red line) shows a weak partial agonist effect, **16 b** (blue line) behaves as an antagonist. Both effects were confirmed when **9 b** (red dots) and **16 b** (blue dots) completely inhibited the agonist activity of DAMGO (EC_80_ concentration of 30 nM for IP and 1 μM for arrestin). B, D) Arrestin recruitment was measured by a luminescence‐based enzyme complementation assay in HEK‐293 cells stably expressing the enzyme acceptor (EA)‐tagged β‐arrestin‐2 fusion protein and the transiently transfected ProLink‐tagged μ‐OR‐PK1. Both **9 b** and **16 b** show neutral antagonist properties (lines) and a strong inhibitory effect on an EC_80_ concentration of DAMGO (dotted line).

Functional assays at μ‐OR were performed with the selected test compounds **9 b** and **16 b** applying an inositol phosphate (IP) accumulation assay for G‐protein mediated signaling (IP‐One assay®) and an arrestin recruitment assay for receptor stimulated recruitment of β‐arrestin‐2 (PathHunter assay). In both signaling pathways the cinnamide **16 b** revealed as a neutral antagonist while the azocarboxamide **9 b** showed antagonist properties for arrestin recruitment but a weak partial agonist effect with an efficacy of 19 % for G‐protein mediated signaling (Figure 2).

These antagonist/partial agonist results could be confirmed by inhibition experiments when the activity of a fixed concentration of the reference agonist DAMGO was fully diminished dose‐dependently by both **9 b** and **16 b** (Figure 2). The resulting IC_50_ values (for **9 b**: 20±6.3 nM for IP accumulation, 22±5.3 nM for arrestin recruitment; for **16 b**: 9.2±5.3 nM for IP, 23±3.2 nM for arrestin) are in good agreement with the observed *K*_i_ values from the binding experiments.

Due to the potential reactivity of the unsaturated azocarboxamide substructure of **9 b** and the cinnamide entity of **16 b** we investigated both compounds on cytotoxicity in comparison to the μ‐OR reference naloxone. For that we incubated HEK293T cells with 100 nM of **9 b**, **16 b** or naloxone for 24 hours and determined the number of cells indicating any influence on cell growth resulting in cell densities of 92±6 % (*n*=6, mean±SEM) for **9 b**, 106±6 % (*n*=6) for **16 b** and 84±5 % (*n*=6) for naloxone relative to the effect of vehicle (DMSO). Complemented by the verification of viability of the cells by optical controls these results reveal no cytotoxic effect of the ligands.

In the next step, the binding modes of the ligands **5**, **9 a**–**d**, **16 a** and **16 b** within the μ‐OR, the κ‐OR the δ‐OR subtype were compared (Figures S5–S7 in the Supporting Information) by using available X‐ray structures (PDB IDs μ‐OR: 4DKL; δ‐OR: 4 N6H; κ‐OR: 4DJH) as templates. All three structures were obtained by co‐crystallization with antagonists, which ensures that the receptor geometries represent the inactive state and are therefore directly comparable by geometric superposition. Within the μ‐OR subtype, the morphinan scaffold of the ligands **5**, **9 a**–**9 d**, **16 a** and **16 b** adopts the same orientation, whereas the side chain flips depending on the attached substituents, in particular for **9 c** and **9 d** bearing larger substituents on the azocarboxamide (Figure S3). The preferred binding modes found for **5**, **9 a**–**9 d**, **16 a** and **16 b** in the κ‐OR do not show a significant dependence on the structural variations (Figure S4). In comparison to the binding modes predicted for the μ‐OR subtype (Figure S3), those at the κ‐OR (Figure S4) however suggest a completely different position of the morphinan scaffold, which could be due to the fact the κ‐OR binding pocket is narrower than those of the μ‐OR and δ‐OR. All ligands **5, 9 a**–**d** and **16 a,b** are also likely to present very similar binding modes within the δ‐OR subtype (Figure S5), whereat the overall orientation of the ligands is comparable to binding mode 2, which was only observed for the two bulkier ligands **9 c** and **9 d** in the μ‐OR subtype (Figure S3).

**Figure 3:**
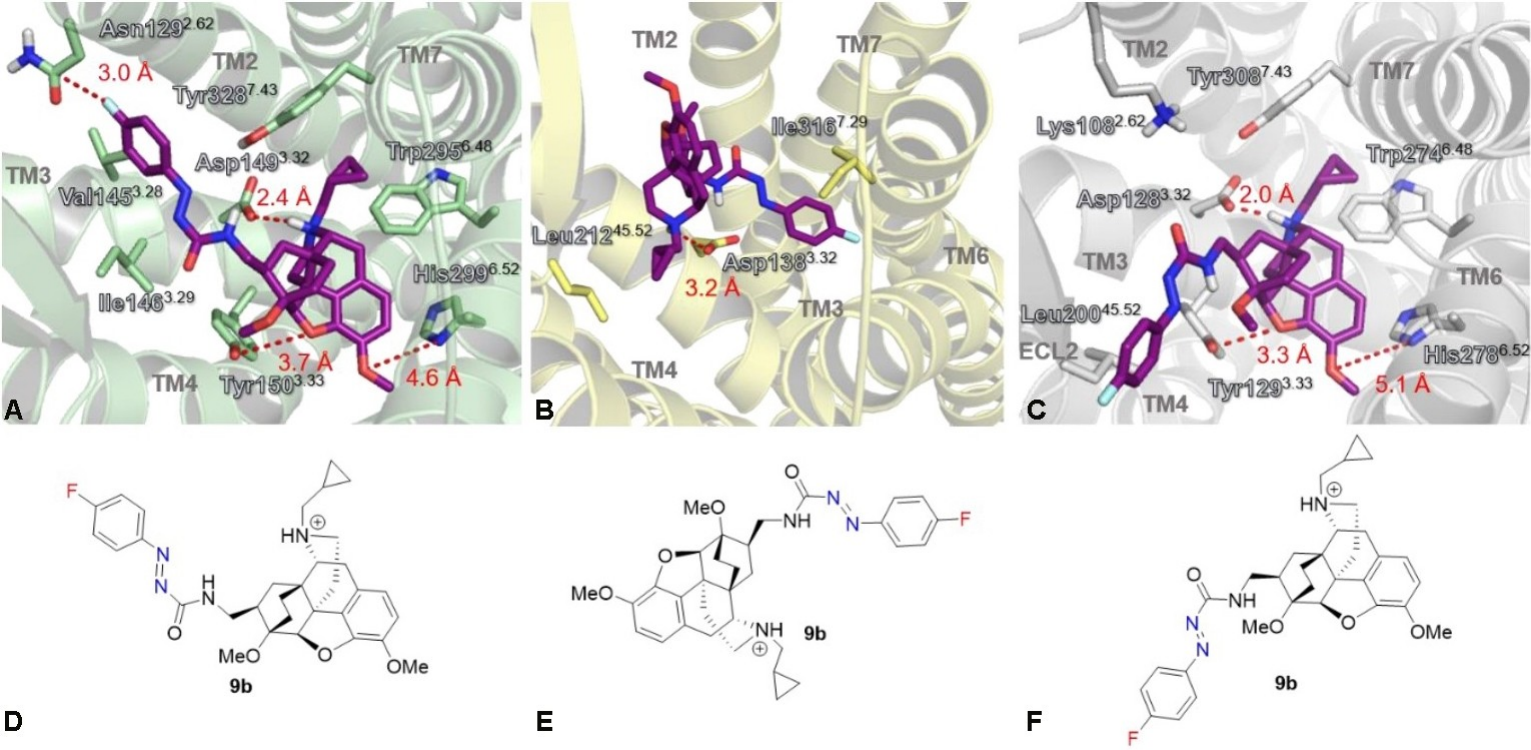
Surroundings of the best‐ranked pose of azocarboxamide **9 b** (purple) in A) the μ‐OR, B) the κ‐OR and C) the δ‐OR subtype. The structures were visualized by using PyMOL 1.3. The poses that are adopted by the **9 b** subtype are shown as Lewis structures in D) the μ‐OR, E) the κ‐OR and F) the δ‐OR subtypes.

The key interactions of azocarboxamide **9 b**, which is one of the two ligands **9 b** and **9 c** with the most favorable binding profile (Table 2), to the μ‐OR, κ‐OR and the δ‐OR subtype were analyzed and are shown in Figure 3.

The interactions of the protonated amine of **9 b** to Asp149^3.32^ (μ‐OR, Figure 3A) and Asp128^3.32^ (δ‐OR, Figure 3C) as well as between the bridging ether oxygen of **9 b** to Tyr150^3.33^ (μ‐OR, Figure 3A) and Tyr129^3.33^ (δ‐OR, Figure 3C) are comparable in both subtypes. Although the distances between the ether oxygen of **9 b** and the hydroxy group on the adjacent Tyr150^3.33^ or Tyr129^3.33^ (3.7 Å for the μ‐OR and 3.4 Å for the δ‐OR) appear as too long for a hydrogen bond, it is important to note that amino acid side chains are not flexible in our docking setup, so that geometric relaxation of the docked complex by energy minimization could indeed enable this interaction. Additionally, the hydrophobic environment is very similar in both subtypes, leading to an almost identical placement of the morphinan scaffold with its cyclopropyl side chain within the binding pocket. Interestingly, the 4‐fluoro substituent on the phenylazocarboxamide side chain is likely to form a σ‐hole‐based halogen bond to the carbonyl unit of Asn129^2.62^ (TM2) within the μ‐OR subtype, which could induce subtype selectivity. The δ‐OR possesses a bulkier Lys108^3.32^ without halogen bond capability at position of Asn129^2.62^ in the μ‐OR subtype. This results in a different binding mode of the flexible 4‐fluorophenyl side chain due to steric restrictions. Within the κ‐OR subtype, the geometry of the binding pocket does not allow an orientation of the morphinan scaffold as in the other subtypes (see also Figure S4). Especially the residues Ile290^6.51^, Ile294^6.55^, and Tyr312^7.35^ are responsible for these geometric restrictions. In the alternative binding mode, compound **9 b** is able to form a hydrogen bond from the CONH group to Asp138^3.32^. With only minor reorientation of this Asp side chain, an additional hydrogen bond to the protonated amine nitrogen is possible, characterizing Asp138^3.32^ as a key residue for the ligand recognition. Despite the different orientation of **9 b** within the other subtypes, the 4‐fluorophenyl side chain finds itself in a stabilizing hydrophobic environment formed by the residues Trp287^6.48^, Ile290^6.51^, Ile316^7.39^, and Tyr320^7.43^. Unfortunately, the bridging oxygen atom of the morphinan scaffold is not directly involved in interaction to the protein. However, this deficiency is compensated by the strong polar interactions of the ligand to Asp138^3.32^.

To get an impression about the metabolic stability of the newly synthesized azocarboxamide and cinnamide ligands, we treated azocarboxamide **9 b** and its cinnamide analogue **16 b** with rat liver microsomes. To enable a largely independent evaluation of the phenylazocarboxamide and the cinnamide motif, the simple piperidine‐derived azocarboxamide **18** and its corresponding cinnamide **19** were also prepared. In Table 3, the determined half‐lives (*t*_1/2_) and intrinsic clearances (*CL’*_int_) of the azocarboxamides and cinnamides are summarized together with the values obtained for the reference compound imipramine (**20**).

**Table 3:** Metabolic stability of azocarboxamides **9 b** and **18** and cinnamides **16 b** and **19** in the presence of rat liver microsomes.

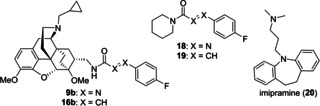		
Compound	Half‐life^[a]^ [min]	Intrinsic clearance^[a]^ [μL×min^−1^×mg^−11^]
**9 b**	166±16	42±4
**16 b**	252±94	28±5
**18**	73±12	97±15
**19**	85±18	85±20
**20**	52±6	132±4

[a] The metabolic stability was determined *via* the half‐life and the intrinsic clearance, which were calculated depending on the amount of rat microsomes that was used (see Supporting Information).

The positive control imipramine (**20**) showed the shortest half‐life (52±6 min) and corresponding to that also the highest clearance with 132±4 μL×min^−1^×mg^−1^ (Table 3). The two piperidine‐derived compounds **18** and **19** displayed slightly higher, but comparable stabilities (**18**: *t*_1/2_=73±12 min; **19**: 85±18 min), thus demonstrating that the bioisosteric replacement of a cinnamide unit by a phenylazocarboxamide does only result in a weakly reduced life‐time under the chosen assay conditions. For each compound **18** and **19**, LC/MS analysis of the reaction mixture revealed the formation of two oxidized species, whereat the mass difference of *m*/*z*+16 points to hydroxylation or, is the case of the cinnamide, possibly also to epoxidation. The trend that the cinnamide derivative shows a higher metabolic stability than the corresponding phenylazocarboxamide also turned out to be true for the ligands **16 b** and **9 b**, albeit with larger relative deviation than previously found for **18** and **19**. For the azocarboxamide **9 b** displaying the favorable binding profile (Table 2), a half‐life of 166±16 min and an intrinsic clearance of 42±4 μL min^−1^ mg^−1^ were determined, suggesting sufficient metabolic stability of [^18^F]**9b** for *in‐vivo* use as a PET imaging agent. The metabolite which was primarily detected by LC/MS for **9 b** is likely to be the N‐dealkylated species (*m*/*z*−54, cleavage of cyclopropylmethyl group) along with small amounts of a reduced derivative (*m*/*z*+2) that could result from reduction of the azo unit to the corresponding hydrazine. For the cinnamide analogue **16 b** showing the slightly higher metabolic stability (*t*_1/2_=252±94 min, *CL’*_int_=28±5 μL min^−1^ mg^−1^), only one metabolite corresponding to N‐dealkylation (*m*/*z*−54) was found. Interestingly, oxidized species (*m*/*z*+16), as they were observed as main metabolites for **18** and **19**, were neither detected for **9 b** nor for **16 b**.

**Scheme 3:**
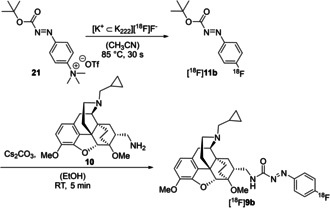
Radiosynthesis of the ^18^F‐labeled azocarboxamide [^18^F]**9b**.

The radiosynthesis shown in Scheme 3 takes advantage of the excellent availability of the ^18^F‐labeled *tert*‐butyl phenylazocarboxylate [^18^F]**11b**, which can readily be prepared in a single step from the quaternary ammonium triflate **21** in only 30 seconds and in high radiochemical yield.^41^ Based on our previously developed radiosyntheses,^40^, ^41^, ^42^, ^43^ we herein studied the coupling of [^18^F]**11b** to the primary amine **10**, aiming at an optimum radiochemical yield of [^18^F]**9b**.

Nucleophilic substitution of azo ester [^18^F]**11b** with the primary amine **10** in ethanol at room temperature in the presence of cesium carbonate afforded [^18^F]**9b** within a short reaction time of only 5 minutes, whereat the identity of [^18^F]**9b** was confirmed through co‐injection with the reference compound **9 b** by HPLC methods (see Supporting Information). A concentration of amine **10** of 54 mM led to a radiochemical yield of [^18^F]**9b** of 11±2 % (*n*=3) after 5 minutes reaction time, which did not increase any further after 10 minutes. The concentration of amine **10** was therefore increased to 108 mM, which led to an only slightly higher radiochemical yield of 13±2 % (*n*=3) after 5 minutes. The relatively low radiochemical yield of the second reaction step could be due to the formation of [^18^F]fluorobenzene as a major byproduct and has kept us from further experiments with [^18^F]**9b**. Nevertheless, [^18^F]**9b** was proven to be stable under the reaction conditions and could be successfully isolated by HPLC in radiochemically pure form. Provided that the radiosynthesis could be automated using a synthesis module, [^18^F]**9b** could be made available by the use of *tert*‐butyl 4‐[^18^F]fluorophenyl‐azocarboxylate [^18^F]**11b** in larger amounts for further characterization by *in vitro* rat brain autoradiography or extended *in vivo* small animal PET imaging studies. As the opioid receptor, especially the μ subtype, is of high interest in the field of brain research concerned with addiction or pain processing,^48^ subtype selective μ‐OR radioligands for imaging studies by positron emission tomography (PET) are valuable tools to study the regulation of the μ subtype in vivo. The currently available ^11^C‐labeled OR receptor ligands for PET, such as ^11^C‐carfentanil or ^11^C‐PEO,^44^, ^49^ suffer from the short half‐life (20.3 min) and their use is clearly restricted to institutions with cyclotrons. The radiosyntheses of most ^18^F‐labeled OR ligands, such as ^18^F−FE−DPN or ^18^F−FE−PEO,^46^, ^50^ are rather laborious and give low yields. Therefore, an alternative ^18^F‐labeled μ‐OR ligand, available by a more straightforward radiosynthesis is still desirable.

## Conclusion

In summary, a novel type of buprenorphine‐derived opioid receptor ligands was obtained upon targeted structural modification. Besides the introduction of a methyl group on the 3‐hydroxy functionality of the buprenorphine core, the cinnamide side chain of the lead structure **5** was exchanged for various phenylazocarboxamide moieties, also with the aim to explore future suitability for ^18^F‐radiosynthesis. Ligand binding studies at the three OR subtypes μ, κ and δ revealed an even increased subtype selectivity for ligands **9** compared to **5** at a basically unchanged affinity to the μ subtype. Evaluation of the azocarboxamide **9 b** in functional assays showed a weak bias for **9 b**, as this compound acts as an antagonist in the β‐arrestin pathway, but as a weak partial agonist in G protein activation. Its cinnamide analogue **16 b**, in contrast, behaved as a neutral antagonist in both pathways. Docking studies gave an impression on the possible binding modes thereby supporting the experimental observation that methylation at the 3‐hydroxy group of the buprenorphine core is not decisive for ligands of type **9** or **16**. Further assays revealed a reasonable metabolic stability of azocarboxamide **9 b** compared to **16 b** and related reference compounds. Finally, the course of the ^18^F‐radiosynthesis of an opioid receptor radioligand candidate for PET was demonstrated by the straightforward synthesis of [^18^F]**9b**.

## Experimental Section

**General experimental**. Reactions which are sensitive to air or water were evacuated under traditional Schlenk conditions while heating and under an inert argon atmosphere. Solvents and reagents for sensitive reactions classified as “extra pure”, “dry” or “extra dry” or with water contents lower than 0.1 % were applied. For reactions which were insensitive towards water, chemicals classified as “pure” or “for synthesis” were used. The applied chemicals were used as purchased by commercial sources (Alfa Aesar, Acros Organics, Merck, Sigma Aldrich). Thin‐layer chromatography (TLC) was performed on purchased plates from Merck (aluminium foil, 0.25 mm Kieselgel 60, F_254_). The substances were determined applying fluorescence detection under ultraviolet light with wavelengths of *λ*=254 and 360 nm [UV]. Liquid flash column chromatography was performed on silica gel with a particle size between 40 and 63 μm (230–400 mesh ASTM, Si 60) from Merck under a pressure between 1.0 and 1.5 bar. The applied eluents are described in the respective procedure, whereby gradients are presented by arrows (→). For mass spectrometry (MS) a Waters Alliance e2695 Separations Module system was used with a Waters 2998 Photodiode Array Detector for detection at wavelength of *λ*=220 nm and 254 nm. A binary solvent gradient of 0.1 % formic acid in acetonitrile/0.1 % formic acid in water was applied on a Waters XBridge C_18_ (4.6 mm×50 mm, 3.5 μm). The runs for the measuring of the metabolites for metabolism studies were performed on a longer Phenomenex Gemini NX‐C18 (110 Å, 4.6×250 mm, 3.5 μm) column. Masses were detected by electro spray ionization (ESI) in a Waters Acquity QDa detector. For ESI‐TOF high mass accuracy and resolution experiments an AB Sciex Triple TOF660 SCiex or a Bruker maXis MS was applied. High‐performance liquid chromatography (HPLC) was used for purification by preparative RP‐HPLC was performed on Agilent 1100 Preparative Series which is equipped with a VWD detector with wavelengths of λ=230 nm and 254 nm. A column from Macherey‐Nagel Varioprep VP 250/32 Nucleodur C_18_ HTec with 5 μm particles [C_18_] with a flow rate of 32 mL/min was applied. NMR spectra were recorded with Avance 600 (^1^H NMR: 600 MHz, ^13^C NMR: 151 MHz) and Avance 360 (^1^H NMR: 400 MHz, ^13^C NMR: 101 MHz) instruments from Bruker at 300 K in deuterated solvents from Deutero GmbH. The chemical shifts *δ* are presented in ppm and are calibrated either in accordance to tetramethylsilane (TMS) or to the used deuterated solvents:^51^ Coupling constants *J* are listed as experimentally determined differences of the frequencies. Coupling between fluoro and carbon atoms in ^13^C spectra are presented as *J*_CF_. The determination of the spectra was performed using Mestre‐C and TopSpin.

***N*** **‐Northebaine (12’)**: Thebaine (**12**, 550 mg, 1.77 mmol) was dissolved in chloroform/isopropanol (3 : 1, 40 mL) and subsequently *meta*‐chloroperoxybenzoic acid (407 mg (75 % content), 1.77 mmol) was added at −5 °C. After 15 minutes of stirring, the mixture was treated with hydrochloric acid (36 m, 156 μL) and ferrocene acetic acid (57 mg, 12.5 mol%) and stirred at 50 °C for further 28 h. After cooling to RT, aqueous sodium hydroxide solution (5 %, 15 mL) was added and the crude product **12’** was extracted with chloroform (3×50 mL). The combined organic layers were washed with saturated aqueous sodium chloride solution and dried over anhydrous sodium sulfate. The solvent was removed under reduced pressure. Afterwards, the crude product **12’** was purified by column chromatography (deactivated with CH_2_Cl_2_, then 40 : 1 : 0.4→30 : 1 : 0.4 CH_2_Cl_2_/MeOH/NH_3_ (25 %)). The desired product **12’** was obtained as gray solid (421 mg, 1.42 mmol, 80 %). *R*_f_=0.3 (35 : 1 : 0.4 CH_2_Cl_2_/MeOH/NH_3_ (25 %) [ninhydrin]. ^1^H NMR (600 MHz, CDCl_3_) δ=6.67 (d, *J*=8.2 Hz, 1H), 6.61 (d, *J*=8.1 Hz, 1H), 5.48 (d, *J*=6.4 Hz, 1H), 5.26 (s, 1H), 5.03 (d, *J*=6.4 Hz, 1H), 3.89 (dd, *J*=5.3, 2.5 Hz, 1H), 3.85 (s, 3H), 3.60 (s, 3H), 3.19 (td, *J*=13.2, 3.5 Hz, 1H), 3.15–3.08 (m, 2H), 2.91 (dd, *J*=13.8, 4.1 Hz, 1H), 2.06 (td, *J*=12.6, 5.1 Hz, 1H), 1.82 (dt, *J*=16.1, 8.1 Hz, 1H), 1.59 (bs, 3H) ppm. ^13^C NMR (91 MHz, CDCl_3_) δ=152.6 (C_q_), 144.9 (C_q_), 142.9 (C_q_), 133.5 (C_q_), 127.9 (C_q_), 119.3 (CH), 113.0 (CH), 110.2 (CH), 95.9 (CH), 89.3 (CH), 56.5 (CH_3_), 55.0 (CH_3_), 54.0 (CH_3_), 46.8 (CH_2_), 40.8 (C_q_), 38.6 (CH_2_), 37.9 (CH_2_) ppm.

***N*** **‐(Cyclopropylmethyl)northebaine (13)**: *N*‐Northebaine (**12’**, 266 mg, 0.89 mmol), sodium carbonate (300 mg, 3.58 mmol) and (bromomethyl)cyclopropane (133 mg, 96 μL, 0.98 mmol) were dissolved in DMF (4.5 mL). The resulting mixture was stirred for 20 h at 90 °C. After cooling to RT, water was added and the crude product was extracted with chloroform (3×75 mL). The combined organic layers were dried over sodium sulfate. After the solvent was removed under reduced pressure, the crude product was purified by column chromatography (49 : 1→30 : 1 CH_2_Cl_2_/MeOH). The desired product **13** was obtained as light brown solid (281 mg, 0.80 mmol, 90 %). *R*_f_=0.5 (20 : 1 CH_2_Cl_2_/MeOH) [ninhydrin]. ^1^H NMR (600 MHz, CDCl_3_) δ=6.66 (d, *J*=8.2 Hz, 1H), 6.60 (d, *J*=8.2 Hz, 1H), 5.58 (d, *J*=6.4 Hz, 1H), 5.29 (d, *J*=4.8 Hz, 1H), 5.04 (d, *J*=6.4 Hz, 1H), 3.96 (d, *J*=6.5 Hz, 1H), 3.85 (s, 3H), 3.60 (s, 3H), 3.30 (d, *J*=17.8 Hz, 1H), 2.93 (dt, *J*=17.2, 8.4 Hz, 1H), 2.87 (dd, *J*=20.8, 6.1 Hz, 1H), 2.75 (dd, *J*=17.9, 7.0 Hz, 1H), 2.53 (d, *J*=6.3 Hz, 2H), 2.21 (td, *J*=12.6, 5.2 Hz, 1H), 1.73 (dd, *J*=12.7, 1.9 Hz, 1H), 1.00–0.91 (m, 1H), 0.62–0.53 (m, 2H), 0.21–0.14 (m, 2H) ppm. ^13^C NMR (151 MHz, CDCl_3_) δ (ppm) 152.8 (C_q_), 144.8 (C_q_), 143.0 (C_q_), 133.6 (C_q_), 127.7 (C_q_), 119.4 (CH), 113.1 (CH), 96.0 (CH), 89.3 (CH), 59.0 (CH_2_), 58.8 (CH), 56.6 (CH_3_), 55.1 (CH_3_), 46.6 (C_q_), 44.3 (CH_2_), 36.6 (CH_2_), 30.9 (CH_2_), 9.4 (CH), 4.1 (CH_2_), 4.0 (CH_2_) ppm.

**Ethyl 17‐(cyclopropylmethyl)‐4,5α‐epoxy‐3,6‐dimethoxy‐6,14‐endoethenylene‐morphinan‐7α‐carboxylate** (**14**): *N*‐(Cyclopropyl‐methyl)northebaine (13, 241 mg, 0.68 mmol) was dissolved in ethyl acrylate (1.84 g, 2.00 mL, 18.4 mmol) and the mixture was stirred for 15 h at 100 °C. After completion of the reaction, the excess of ethyl acrylate was removed under reduced pressure. The crude product **14** was purified by column chromatography (40 : 1 CH_2_Cl_2_/MeOH). The desired product **14** was obtained as brown solid (266 mg, 0.59 mmol, 87 %). *R*_f_=0.5 (35 : 1 CH_2_Cl_2_/MeOH) [CAM]. ^1^H NMR (600 MHz, CDCl_3_) δ=0.10–0.16 (m, 2H), 0.47–0.54 (m, 2H), 0.79–0.86 (m, 1H), 1.25 (t, *J=*7.1 Hz, 3H), 1.47 (dd, *J=*6.4, 12.7 Hz, 1H), 1.82–1.87 (m, 1H), 1.97 (dt, *J=*5.5, 12.6 Hz, 1H), 2.32–2.45 (m, 4H), 2.70 (dd, *J=*4.9, 12.1 Hz, 1H), 2.82–2.86 (m, 1H), 3.08–3.12 (m, 2H), 3.54 (d, *J=*6.5 Hz 1H), 3.62 (s, 3 H), 3.82 (s, 3H), 4.09–4.19 (m, 2H), 4.60 (d, *J=*1.4 Hz, 1H), 5.56 (d, *J=*8.8 Hz, 1H), 5.84 (d, *J=*8.8 Hz, 1H), 6.52 (d, *J=*8.1 Hz, 1H), 6.62 (d, *J=*8.1 Hz, 1H) ppm. ^13^C NMR (91 MHz, CDCl_3_) δ=173.6 (C_q_), 148.2 (C_q_), 142.0 (C_q_), 135.6 (CH), 134.5 (C_q_), 128.4 (C_q_), 126.6 (CH), 119.5 (CH), 113.6 (CH), 94.1 (CH), 81.2 (C_q_), 60.7 (CH_2_), 59.9 (CH_2_), 57.2 (CH), 56.8 (CH_3_), 53.0 (CH_3_), 48.1(C_q_), 44.2 (CH_2_), 43.4 (CH), 43.1 (C_q_), 33.8 (CH_2_), 31.1 (CH_2_), 23.4 (CH_2_), 14.4 (CH_3_), 9.6 (CH), 4.2 (CH_2_), 3.6 (CH_2_) ppm.

**17‐(Cyclopropylmethyl)‐4,5α‐epoxy‐3,6‐dimethoxy‐6,14‐endoethenylenemorphinan‐7α‐caboxylic acid hydrochloride** (**14’**): A solution of the ester 14 (266 mg, 0.59 mmol) in hydrochloric acid (6 M) was stirred for 6 h at 105 °C. After cooling to RT, the hydrochloric acid was removed under reduced pressure. The crude product **14’** (260 mg, 0.57 mmol, 96 %) was used for the next step without further purification. ^1^H NMR (600 MHz, (CD_3_)_2_SO) δ=12.25 (s, 1H), 8.73 (s, 1H), 6.74 (d, *J*=8.2 Hz, 1H), 6.61 (d, *J*=8.2 Hz, 1H), 5.65 (d, *J*=8.8 Hz, 1H), 5.57 (d, *J*=8.8 Hz, 1H), 4.93 (s, 1H), 4.42 (d, *J*=6.7 Hz, 1H), 3.72 (s, 3H), 3.46 (d, *J*=7.0 Hz, 3H), 3.45–3.39 (m, 3H), 3.14–2.91 (m, 5H), 2.26 (td, *J*=14.5, 5.1 Hz, 1H), 3.45–3.39 (m, 1H) 1.48 (dt, *J*=14.8, 7.4 Hz, 1H), 1.15–1.05 (m, 1H), 0.86 (dd, *J*=8.4, 5.5 Hz, 1H), 0.77–0.64 (m, 2H), 0.61–0.41 (m, 2H) ppm.

***N*** **‐Benzyl‐17‐(cyclopropylmethyl)‐4,5α‐epoxy‐3,6‐dimethoxy‐6,14‐ethenylenemorphinan‐7α‐carboxamide (15)**: A solution of carboxylic acid **14’** (216 mg, 0.47 mmol) in dry chloroform (8.5 mL) was cooled to 0 °C and treated with oxalyl chloride (907 mg, 0.61 mL, 7.14 mmol). The resulting mixture was stirred at RT for 23 h. Afterwards, the solvent was removed under reduced pressure. The remaining crude acid chloride **14’’** was dissolved in dry chloroform (7 mL). Benzylamine (474 mg, 0.48 mL, 4.42 mmol) and triethylamine (352 mg, 0.48 mL, 3.48 mmol) were added at 0 °C and the reaction mixture was stirred at RT for 22 h. After completion of the reaction, the mixture was filtered, and the filtrate was concentrated under reduced pressure. The crude product **15** was purified by column chromatography (200 : 1 : 2→150 : 1 : 2→100 : 1 : 2 CH_2_Cl_2_/MeOH/ NH_3_(25 %)). The desired product **15** was obtained as brown solid (191 mg, 0.37 mmol, 79 %). *R*_f_=0.5 (20 : 1 CH_2_Cl_2_/MeOH) [UV]. ^1^H NMR (400 MHz, CDCl_3_) δ=7.37–7.27 (m, 5H), 6.62 (d, *J*=8.1 Hz, 1H), 6.52 (d, *J*=8.1 Hz, 1H), 6.39 (t, *J*=5.5 Hz, 1H), 5.90 (d, *J*=8.8 Hz, 1H), 5.59 (d, *J*=8.8 Hz, 1H), 4.56 (d, *J*=1.4 Hz, 1H), 4.52–4.37 (m, 2H), 3.81 (s, 3H), 3.61 (s, 3H), 3.59–3.55 (m, 1H), 3.17 (dd, *J*=13.2, 9.7 Hz, 1H), 3.14–3.07 (m, 1H), 2.76–2.65 (m, 2H), 2.47–2.29 (m, 4H), 2.00 (td, *J*=12.6, 4.9 Hz, 1H), 1.84 (dd, *J*=13.0, 2.3 Hz, 2H), 1.60 (dd, *J*=13.3, 6.2 Hz, 1H), 0.89–0.79 (m, 1H), 0.54–0.47 (m, 2H), 0.13 (d, *J*=4.9 Hz, 2H) ppm. ^13^C NMR (91 MHz, CDCl_3_) δ=172.5 (C_q_), 148.0 (C_q_), 141.9 (C_q_), 138.6 (C_q_), 137.3 (CH), 134.4 (C_q_), 128.6 (2×CH), 128.3 (C_q_), 127.5 (2×CH), 127.3 (CH), 125.6 (CH), 119.5 (CH), 113.5 (CH), 94.9 (CH), 80.7 (C_q_), 59.8 (CH_2_), 57.1 (CH), 56.6 (CH_3_), 53.1 (CH_3_), 47.9 (C_q_), 44.9 (CH), 44.0 (CH_2_), 43.6 (CH_2_), 43.0 (C_q_), 33.6 (CH_2_), 30.8 (CH_2_), 23.2 (CH_2_), 9.5 (CH), 4.2 (CH_2_), 3.4 (CH_2_) ppm.

***N*** **‐Benzyl‐17‐(cyclopropylmethyl)‐4,5α‐epoxy‐3,6‐dimethoxy‐6,14‐ethenylenemorphinan‐7α‐ methanoamine (15’)**: Amide **15** (212 mg, 0.413 mmol) was dissolved in dry THF (1.5 mL) and subsequently added to a suspension of lithium aluminum hydride (2.4 m in THF, 1.10 mmol, 0.45 mL). The reaction mixture was stirred for 20 h under reflux. After completion of the reaction, an aqueous sodium sulfate was slowly added, and the precipitating solid was removed by filtration. The filtrate contained the crude product **15’** which was purified by column chromatography (100 : 1 : 1 CH_2_Cl_2_/MeOH/NH_3_(25 %)). The desired product **15’** was obtained as yellow oil (127 mg, 0.26 mmol, 62 %). *R*_f_=0.5 (100 : 3 : 1 CH_2_Cl_2_/MeOH/ NH_3_(25 %)) [KMnO_4_]. ^1^H NMR (600 MHz, CDCl3) δ=7.34–7.30 (m, 4H), 7.26–7.22 (m, 1H), 6.60 (d, *J*=8.1 Hz, 1H), 6.49 (d, *J*=8.1 Hz, 1H), 5.72 (d, *J*=8.7 Hz, 1H), 5.45 (d, *J*=8.8 Hz, 1H), 4.59 (d, *J*=1.3 Hz, 1H), 3.81 (s, 3H), 3.78 (s, 1H), 3.56 (s, 3H), 3.45 (d, *J*=6.6 Hz, 1H), 3.11–3.01 (m, 2H), 2.77 (dd, *J*=11.8, 6.2 Hz, 1H), 2.70 (dd, *J*=12.0, 4.9 Hz, 1H), 2.42–2.35 (m, 3H), 2.35–2.29 (m, 1H), 2.29–2.23 (m, 1H), 2.15–2.09 (m, 1H), 1.99 (td, *J*=12.7, 5.5 Hz, 1H), 1.81 (dd, *J*=13.0, 2.3 Hz, 1H), 0.93–0.76 (m, 4H), 0.55–0.47 (m, 2H), 0.17–0.08 (m, 2H) ppm.

**7α‐(Aminomethyl)‐17‐(cyclopropylmethyl)‐4,5α‐epoxy‐3,6‐dimethoxy‐6,14‐ethane‐morphinan (10)**: Compound **15’** (160 mg, 0.32 mmol) and ammonium formate (121 mg, 1.92 mmol) were dissolved in absolute ethanol (5 mL) and the resulting mixture was treated with palladium on carbon (10 %, 34.0 mg, 319 μmol). The reaction mixture was stirred for 1.5 h under reflux. Afterwards, the remaining palladium on carbon was removed by filtration over *Celite* and the solvent was removed under reduced pressure. The crude product was purified by column chromatography (20 : 1 : 0.2 CH_2_Cl_2_/MeOH/NH_3_(25 %)) yielding amine **10** as yellow oil (106 mg, 0.26 mmol, 81 %). *R*_f_=0.5 (91 : 7.5 : 1 CH_2_Cl_2_/MeOH/ NH_3_(25 %)) [KMnO_4_]. ^1^H NMR (360 MHz, CDCl_3_) δ=6.70 (d, *J*=8.1 Hz, 1H), 6.55 (d, *J*=8.1 Hz, 1H), 4.50 (d, *J*=1.6 Hz, 1H), 3.87 (s, 3H), 3.42 (s, 3H), 3.03–2.95 (m, 3H), 2.65 (dd, *J*=11.8, 5.2 Hz, 1H), 2.37–2.20 (m, 5H), 2.05 (td, *J*=12.7, 5.6 Hz, 1H), 1.90 (s), 1.66 (dd, *J*=12.9, 2.5 Hz, 1H), 1.58–1.36 (m, 3H), 1.24–1.19 (m, 1H), 1.11–0.97 (m, 2H), 0.79–0.68 (m, 2H), 0.52–0.45 (m, 2H), 0.13–0.06 (m, 2H) ppm. ^13^C NMR (151 MHz, CDCl_3_) δ=147.0, 141.8, 132.8, 128.7, 119.0, 113.8, 93.0, 60.0, 58.7, 56.7, 51.2, 45.8, 43.8, 43.7, 42.7, 38.3, 35.6, 35.5, 33.4, 29.3, 22.8, 18.8, 9.4, 4.0, 3.6 ppm. HRMS (ESI) calcd for C_25_H_35_N_2_O_3_ [*M*+H]^+^: 411.2642, found: 411.2647

***N*** **‐(Phenyl)‐17‐(cyclopropylmethyl)‐4,5α‐epoxy‐3,6‐dimethoxy‐6,14‐ethanemorphinan‐7α‐yl‐methyl)azocarboxamide (9 a)**: Amine **10** (51.0 mg, 0.12 mmol), *tert*‐butyl (*E*)‐2‐phenyldiazene‐1‐carboxylate (**11 a**) (51.3 mg, 0.24 mmol) and potassium carbonate (85.7 mg, 0.62 mmol) were dissolved in dry ethanol (3 mL) and stirred at RT for 3 d. After addition of water, the reaction mixture was extracted with ethyl acetate (3×20 mL) and dried over sodium sulfate. The crude product was purified by column chromatography (100 : 1 : 1 CH_2_Cl_2_/MeOH/NH_3_(25 %)) yielding **9 a** as yellow powder (48.0 mg, 0.09 mmol, 71 %). ^1^H NMR (400 MHz, CD_3_CN) δ=7.92–7.87 (m, 2H), 7.67–7.57 (m, 3H), 7.18 (bs, 1H), 6.75 (d, *J*=8.1 Hz, 1H), 6.57 (d, *J*=8.1 Hz, 1H), 4.57 (d, *J*=2.1 Hz, 1H), 3.81 (s, 3H), 3.60 (dt, *J*=13.4, 5.1 Hz, 1H), 3.44–3.39 (m, 1H), 3.38 (s, 3H), 3.02–2.89 (m, 3H), 2.66 (dd, *J*=11.8, 5.2 Hz, 1H), 2.33–2.20 (m, 5H), 2.12–2.02 (m, 1H), 1.59 (dd, *J*=13.1, 2.5 Hz, 1H), 1.51–1.41 (m, 1H), 1.35–1.25 (m, 2H), 1.19 (dd, *J*=13.3, 5.8 Hz, 1H), 1.10 (td, *J*=12.6, 6.3 Hz, 1H), 0.80–0.71 (m, 1H), 0.71–0.62 (m, 1H), 0.47–0.42 (m, 2H), 0.12–0.05 (m, 2H) ppm. ^13^C NMR (151 MHz, CD_3_CN) δ=163.2, 152.4, 148.0, 142.7, 134.3, 133.9, 130.5, 130.1, 124.2, 120.2, 114.8, 91.9, 77.6, 60.6, 59.8, 57.1, 50.8, 46.5, 44.4, 43.0, 36.4, 36.2, 35.2, 33.6, 29.8, 23.5, 20.3, 10.2, 4.3, 4.1 ppm. HRMS (ESI) calcd for C_32_H_38_N_4_O_4_ [*M*+H]^+^: 542.2893, found: 543.2968. Purity: 99 %.

***N*** **‐(4‐Fluorophenyl)‐(17‐(cyclopropylmethyl)‐4,5α‐epoxy‐3,6‐dimethoxy‐6,14‐ethane‐morphinan‐7α‐yl‐methyl)azocarboxamide** (**9 b**): Amine **10** (14.0 mg, 34.0 μmol), *tert*‐butyl (*E*)‐2‐(4‐fluorophenyl)diazene‐1‐carboxylate (**11 b**) (33.6 mg, 0.15 mmol) and potassium carbonate (23.6 mg, 0.17 mmol) were dissolved in dry ethanol (0.15 mL). The reaction mixture was stirred at RT for 2.5 h. After the addition of water, the crude product was extracted with ethyl acetate (3×20 mL) and the combined organic layers were dried over anhydrous sodium sulfate. Afterwards, the crude product was purified by preparative HPLC (5 : 95→100 : 0 MeOH/H_2_O+0.1 % TFA over 30 min, flow rate: 20 mL/min) to give **9 b** as yellow powder (16.5 mg, 29.4 μmol, 87 %). ^1^H NMR (600 MHz, CDCl_3_) δ=10.54 (bs, 1H), 8.04 (dd, *J*=8.7, 5.2 Hz, 2H), 7.22 (t, *J*=8.4 Hz, 2H), 6.84 (d, *J*=8.2 Hz, 1H), 6.70 (d, *J*=8.2 Hz, 1H), 4.66 (s, 1H), 3.93 (s, 3H), 3.90 (d, *J*=5.9 Hz, 1H), 3.77 (d, *J*=13.1 Hz, 1H), 3.67–3.53 (m, 2H), 3.50 (s, 3H), 3.35–3.22 (m, 2H), 3.11 (d, *J*=19.2 Hz, 1H), 3.05–2.99 (m, 1H), 2.91 (dd, *J*=19.0, 5.8 Hz, 1H), 2.86–2.79 (m, 1H), 2.38 (s, 1H), 1.95 (d, *J*=12.0 Hz, 1H), 1.67–1.58 (m, 2H), 1.55–1.46 (m, 1H), 1.42–1.33 (m, 1H), 1.28 (s, 1H), 1.23–1.14 (m, 1H), 0.96–0.88 (m, 1H), 0.85–0.76 (m, 2H), 0.58–0.49 (m, 1H), 0.46–0.38 (m, 1H) ppm. ^13^C NMR (151 MHz, CDCl_3_) δ=168.7 (d, *J*=256.0 Hz), 165.2, 147.4, 143.2, 130.0, 126.5 (d, *J*=9.8 Hz, 2×C), 122.9, 119.8, 116.4 (d, *J*=23.2 Hz, 2×C), 115.4, 90.5, 75.57, 59.7, 59.1, 56.7, 51.0, 45.4, 44.2, 42.2, 35.8, 34.3, 31.9, 31.4, 29.7, 29.2, 24.6, 18.6, 5.6, 5.4, 3.8 ppm. ^19^F NMR (339 MHz, CDCl_3_) δ=−108.1 ppm. HRMS (ESI) calcd for C_32_H_38_FN_4_O_4_ [*M*+H]^+^: 561.2872, found: 561.2866. Purity: 99 %.

***N*** **‐(4‐Bromophenyl)‐(17‐(cyclopropylmethyl)‐4,5α‐epoxy‐3,6‐dimethoxy‐6,14‐ethane‐morphinan‐7α‐yl‐methyl)azocarboxamide** (**9 c**): To a solution of amine **10** (19.5 mg, 0.048 mmol) in dry ethanol (0.4 mL), *tert*‐butyl (*E*)‐2‐(4‐bromophenyl)diazene‐1‐carboxylate (**11 c**) (27.1 mg, 0.10 mmol) in dry ethanol (0.6 mL) and triethylamine (13.2 μL, 0.10 mmol) were added. After the reaction mixture was stirred for 5 d at RT, the solvent was removed under reduced pressure. The crude product was purified by preparative HPLC (5 : 95→100 : 0 MeOH/H_2_O+0.1 % TFA over 30 min, flow rate: 20 mL/min) to give **9 c** (12.5 mg, 0.02 mmol, 42 %) as yellow oil. ^1^H NMR (600 MHz, CD3CN) δ=9.45 (bs, 1H), 7.82 (d, *J*=8.0 Hz, 2H), 7.78 (d, *J*=7.4 Hz, 2H), 7.46 (bs, 1H), 6.88 (d, *J*=8.2 Hz, 1H), 6.71 (d, *J*=8.2 Hz, 1H), 4.73 (d, *J*=2.1 Hz, 1H), 3.89 (d, *J*=8.7 Hz, 1H), 3.85 (s, 3H), 3.65–3.59 (m, 1H), 3.43–3.40 (m, 1H), 3.38 (s, 3H), 3.38–3.34 (m, 1H), 3.21 (d, *J*=19.7 Hz, 1H), 3.16–3.02 (m, 3H), 2.91–2.82 (m, 2H), 1.93–1.89 (m, 1H), 1.58–1.52 (m, 1H), 1.49 (dd, *J*=13.2, 5.2 Hz, 1H), 1.40–1.26 (m, 3H), 1.15–1.06 (m, 1H), 0.79–0.70 (m, 2H), 0.67–0.61 (m, 1H), 0.50–0.45 (m, 1H), 0.42–0.35 (m, 1H) ppm. ^13^C NMR (151 MHz, CD3CN) δ=148.2, 143.5, 133.7, 131.4, 128.3, 125.9, 125.8, 120.9, 118.2, 115.8, 90.1, 76.6, 59.8, 59.6, 57.0, 50.8, 46.0, 45.1, 42.5, 36.6, 32.5, 32.1, 30.8, 29.60, 3.13, 19.9, 6.4, 5.5, 3.8 ppm (one signal is missing due to overlap with solvent signals). HRMS (ESI) calcd for C_32_H_38_BrN_4_O_4_ [*M*+H]^+^: 621.2071, found: 621.2068.

***N*** **‐(4‐Methoxyphenyl)‐(17‐(cyclopropylmethyl)‐4,5α‐epoxy‐3,6‐dimethoxy‐6,14‐ethane‐morphinan‐7α‐yl‐methyl)azocarboxamide** (**9 d**): *tert*‐Butyl (*E*)‐2‐(4‐methoxyphenyl)‐diazene‐1‐carboxylate (**11 d**) (8.90 mg, 0.04 mmol) and triethylamine (5.2 μL, 0.04 mmol) dissolved in dry ethanol (0.3 mL) were added to a solution of amine **10** (7.70 mg, 0.02 mmol) in dry ethanol (0.2 mL). After the reaction mixture was stirred for 5 d at RT, the solvent was removed under reduced pressure. The crude product was purified by preparative HPLC (5 : 95→100 : 0 MeOH/H_2_O+0.1 % TFA over 30 min, flow rate: 20 mL/min) to yield the desired product **9 d** (4.6 mg, 8.0 μmol, 42 %) as yellow oil. ^1^H NMR (600 MHz, CD_3_CN) δ=9.23 (bs, 1H), 7.91 (d, *J=*8.4 Hz, 2H), 7.32 (bs, 1H), 7.11 (d, *J*=7.9 Hz, 2H), 6.88 (d, *J*=8.2 Hz, 1H), 6.71 (d, *J*=8.2 Hz, 1H), 4.73 (d, *J*=2.1 Hz, 1H), 3.90 (s, 3H), 3.88 (d, *J*=7.1 Hz, 1H), 3.85 (s, 3H), 3.63–3.57 (m, 1H), 3.45–3.36 (m, 5H), 3.23–3.18 (m, 1H), 3.15 (dd, *J*=13.4, 7.0 Hz, 1H), 3.06–2.98 (m, 2H), 2.91–2.82 (m, 2H), 1.59–1.45 (m, 2H), 1.40–1.19 (m, 2H), 1.13–1.05 (m, 1H), 0.81–0.70 (m, 2H), 0.68–0.62 (m, 1H), 0.51–0.43 (m, 1H), 0.42–0.35 (m, 1H) ppm (four signals are missing due to overlap with water signals). ^13^C NMR (151 MHz, CD_3_CN) δ=165.1, 148.2, 143.5, 131.4, 126.7 (2×C), 125.8, 120.9, 115.8, 115.6 (2×C), 90.1, 76.7, 59.9, 59.6, 57.0, 56.6, 50.8, 45.9, 45.1, 42.4, 36.6, 34.3, 32.4, 31.9, 30.8, 29.6, 25.1, 20.0, 6.4, 5.4, 3.9 ppm (two signals are missing due to overlap with solvent signals). HRMS (ESI) calcd for C_33_H_40_N_4_O_5_ [*M*+H]^+^: 573.3072, found: 573.3085.

***N*** **‐17‐(cyclopropylmethyl)‐4,5α‐epoxy‐3,6‐dimethoxy‐6,14‐ethane‐morphinan‐7α‐yl‐methyl)cinnamide** (**16 a**): Sodium bicarbonate (32.0 mg, 0.38 mmol) and cinnamoyl chloride (**17 a**) (23.0 mg, 0.14 mmol) were added to amine **10** (10.5 mg, 25.6 μmol) dissolved in dry CH_2_Cl_2_ (1.2 mL). After stirring for two days at RT, water was added, and the crude product was extracted with ethyl acetate (3×20 mL). After the combined organic layers had been dried over sodium sulfate and the solvent had been removed under reduced pressure, the crude product was firstly purified by column chromatography (49 : 1→10 : 1 CH_2_Cl_2_/MeOH) and afterwards by preparative HPLC (5 : 95→100 : 0 MeOH/H_2_O+0.1 % TFA over 28.5 min, flow rate: 20 mL/min). Compound **16 a** was received as yellow oil (3.55 mg, 6.57 μmol, 25 %). ^1^H NMR (600 MHz, CDCl_3_) δ=7.63 (d, *J*=15.0 Hz, 1H), 7.53 (d, *J*=6.9 Hz, 2H), 7.42–7.30 (m, 3H), 7.06–6.97 (m, 1H), 6.82 (d, *J*=8.2 Hz, 1H), 6.67 (d, *J*=8.2 Hz, 1H), 6.64–6.53 (m, 1H), 4.65 (s, 1H), 3.91 (s, 3H), 3.90–3.87 (m, 1H), 3.84–3.80 (m, 1H), 3.68 (d, *J*=12.6 Hz, 1H), 3.53–3.47 (m, 1H), 3.46 (s, 3H), 3.26–3.13 (m, 1H), 3.13–2.99 (m, 2H), 2.94–2.84 (m, 2H), 2.65–2.49 (m, 1H), 2.38–2.26 (m, 1H), 2.02–1.91 (m, 1H), 1.64–1.53 (m, 2H), 1.35–1.22 (m, 2H), 1.22–1.14 (m, 1H), 0.88–0.74 (m, 3H), 0.54–0.47 (m, 1H), 0.44–0.37 (m, 1H) ppm. ^13^C NMR (91 MHz, CDCl_3_) δ=147.6, 143.2, 141.4, 134.9, 132.5, 130.1, 129.6, 128.7, 128.0, 122.9, 119.8, 115.4, 90.3, 75.3, 59.7, 59.2, 56.8, 50.8, 45.7, 44.3, 41.1, 35.8, 34.0, 32.0, 31.6, 29.2, 24.7, 24.6, 18.7, 5.6, 5.5, 3.7 ppm (one signal is missing due to overlap with solvent signals). HRMS (ESI) calcd for C_34_H_41_N_2_O_4_ [*M*+H]^+^: 541.3061, found: 561.3061.

***N*** **‐(4‐Fluorophenyl)‐(17‐(cyclopropylmethyl)‐4,5α‐epoxy‐3,6‐dimethoxy‐6,14‐ethane‐morphinan‐7α‐yl‐methyl)cinnamide** (**16 b**): Amine **10** (15.0 mg, 37 μmol), 4‐fluorocinnamoyl chloride (**17 b**) (34.1 mg, 0.19 mmol) and sodium hydrogen carbonate (46.6 mg, 0.56 mmol) were dissolved in dry ethanol (1.5 mL) and the reaction mixture was stirred at RT for 15 h. After the addition of water, the crude product was extracted with ethyl acetate (3×20 mL) and the combined organic layers were dried over anhydrous sodium sulfate. The solvent was removed under reduced pressure. The crude product was purified by column chromatography (100 : 1 : 1 CH_2_Cl_2_/MeOH/NH_3_(25 %)) and **16 b** (13.0 mg, 23.0 μmol, 63 %) was obtained as gray solid. *R*_f_=0.4 (80 : 1 : 1 CH_2_Cl_2_/MeOH/NH_3_(25 %)) [UV]. ^1^H NMR (600 MHz, CDCl_3_) δ=7.58 (d, *J*=15.6 Hz, 1H), 7.49 (dd, *J*=8.6, 5.4 Hz, 2H), 7.10–7.02 (m, 2H), 6.72 (d, *J*=8.1 Hz, 1H), 6.57 (d, *J*=8.0 Hz, 1H), 6.49 (d, *J*=5.8 Hz, 1H), 6.29 (d, *J*=15.6 Hz, 1H), 4.48 (s, 1H), 3.89 (s, 3H), 3.76–3.69 (m, 1H), 3.51 (s, 3H), 3.41–3.30 (m, 1H), 3.09–2.94 (m, 3H), 2.65 (d, *J*=10.4 Hz, 1H), 2.39–2.20 (m, 4H), 2.12–1.97 (m, 2H), 1.71–1.46 (m, 6H), 1.10–1.02 (m, 1H), 0.84–0.72 (m, 2H), 0.55–0.41 (m, 2H) ppm. ^13^C NMR (151 MHz, CDCl_3_) δ=165.6, 163.6 (d, *J*_CF_=250.1 Hz), 147.0, 142.0, 141.9, 139.6, 132.9, 131.3 (d, *J*=3.4 Hz), 129.7 (2×C, d, *J*_CF_=8.3 Hz), 121.0 (d, *J*=2.2 Hz), 119.3, 116.02 (2×C, d, *J*=21.9 Hz), 114.0, 94.1, 60.1, 58.6, 56.9, 51.9, 46.03, 43.9, 41.9, 36.4, 35.7, 35.5, 33.4, 29.9, 29.3, 22.9, 18.1, 9.6, 4.2, 3.7 ppm. ^19^F NMR (565 MHz, CDCl_3_) δ=‐111.4 ppm. HRMS (ESI) calcd for C_34_H_39_FN_2_O_4_ [*M*+H]^+^: 559.2967, found: 559.2968. Purity: 100 %.

**(*E*)‐((4‐Fluorophenyl)diazenyl)(piperidin‐1‐yl)methanone** (**18**): Phenylazocarboxylic ester **11 b** (112 mg, 0.50 mmol), potassium carbonate (346 mg, 2.50 mmol) and piperidine (0.15 mL, 1.50 mmol) were dissolved in dry ethanol (2 mL). The reaction mixture was stirred for 20 min at 40 °C. After completion of the reaction, diluted hydrochloric acid (3 m) was added, until a pH value of 3 was reached. The crude product **18** was extracted with ethyl acetate (3×50 mL). The combined organic layers were washed with aqueous saturated sodium chloride solution and dried over anhydrous sodium sulfate. After removal of the solvent under reduced pressure, the crude product was purified by column chromatography (3 : 1 hexane/ethyl acetate) yielding **18** (112 mg, 0.48 mmol, 95 %) as orange solid. *R*_f=_0.4 (hexane/ethyl acetate 2 : 1) [UV]. ^1^H NMR (360 MHz, CDCl_3_) δ=1.60–1.66 (m, 2 H), 1.71–1.74 (m, 4 H), 3.57‐3.60 (m, 2 H), 3.72‐3.75 (m, 2 H), 7.20 (dd, *J*_HF_ *=*8.2 Hz, *J=*9.0 Hz, 2 H), 7.95 (dd, *J*_HF_ *=*5.2 Hz, *J=*9.1 Hz, 2 H) ppm. ^13^C NMR (91 MHz, CDCl_3_) δ=24.3 (CH_2_), 25.5 (CH_2_), 26.1 (CH_2_), 44.6 (CH_2_), 46.0 (CH_2_), 116.3 (d, *J*_CF_ *=*23.1 Hz, 2 × CH), 125.8 (d, *J*_CF_ *=*9.4 Hz, 2 × CH), 148.7 (C_q_), 161.4 (C_q_), 165.6 (d, *J*_CF_ *=*255.2 Hz, C_q_). ^19^F NMR (338 MHz, CDCl_3_) δ=−106.4 ppm. HRMS (ESI) calcd for C_12_H_15_FN_3_O [*M*+H]^+^: 236.1190, found: 236.1194

**(*E*)‐3‐(4‐Fluorophenyl)‐1‐(piperidin‐1‐yl)prop‐2‐en‐1‐one** (**19**): (*E*)‐3‐(4‐Fluorophenyl)acryloyl chloride (**17 b**) (50.0 mg, 0.27 mmol) was dissolved in dry CH_2_Cl_2_ (1 mL). Afterwards, the mixture was treated with a solution of piperidine (19.6 mg, 22.0 μL, 0.23 mmol) and triethylamine (31.0 μL, 0.23 mmol) dissolved in ice cold CH_2_Cl_2_ (2 mL). The reaction mixture was stirred at room temperature for 1 h. After completion of the reaction, saturated aqueous sodium bicarbonate solution (10 mL) was added and the crude product **19** was extracted with CH_2_Cl_2_ (3×30 mL). Purification by flash column chromatography (0 %→1 %→5 %→10 % MeOH in CH_2_Cl_2_) gave the desired cinnamide **19** (30.9 mg, 0.17 mmol, 62 %) as a yellow solid. *R*_f_=0.3 (10 : 1 dichloro‐methane/MeOH) [UV]. ^1^H NMR (600 MHz, CDCl_3_) δ=7.63 (d, *J*=15.4 Hz, 1H), 7.56–7.49 (m, 2H), 7.07 (t, *J*=8.6 Hz, 2H), 6.84 (d, *J*=15.4 Hz, 1H), 3.64 (bs, 4H), 1.74–1.67 (m, 2H), 1.67–1.61 (m, 4H) ppm.

**Radioligand binding studies**. Radioligand binding studies with the human opioid receptors μ‐OR, δ‐OR and κ‐OR were performed as described previously.^52^, ^53^ In brief, competition binding experiments were done using membranes of HEK293T cells transiently transfected with the cDNA of the human μ‐OR (gift from the Ernest Gallo Clinic and Research Center, UCSF, CA), δ‐OR and κ‐OR receptor (cDNA Resource Center, Bloomsberg, PA), respectively. Radioligand displacement assays were performed in binding buffer (50 mM Tris, pH 7.4) with [^3^H]diprenorphine (specific activity 31 Ci/mmol, PerkinElmer, Rodgau, Germany) at final concentrations of 0.15–0.50 nM. The assays were carried out at protein concentrations of 1–10 μg/assay tube, a B_max_ value of 3700±730 fmol/μg, a K_D_ value of 0.15±0.026 nM for μ‐OR, 1–10 μg protein/assay tube, B_max_ of 1800±440 fmol/μg, *K*_D_ of 0.24±0.036 nM for δ‐OR, and of 1–6 μg protein/assay tube, *B*_max_ of 6800±2700 fmol/μg, *K*_D_ of 0.12±0.019 nM for κ‐OR respectively. Unspecific binding was determined in the presence of 10 μM naloxone, protein concentration was established by the method of Lowry using bovine serum albumin as standard.^54^ The resulting competition curves of the receptor binding experiments were analyzed by nonlinear regression using the algorithms in PRISM 6.0 (GraphPad Software, San Diego, CA). The data were initially fit using a sigmoid model to provide an IC_50_ value, representing the concentration corresponding to 50 % of maximal inhibition. IC_50_ values were transformed to K_i_ values according to the equation of Cheng and Prusoff.^55^

**Radioligand depletion assay**. Tests on covalent blocking of the receptor were carried out as described previously.^56^ Membranes from HEK 293T cells transiently expressing the human μ‐OR were preincubated in binding buffer at 37 °C at a protein concentration of 100 μg/mL and the test compounds **9 b** and **16 b** at concentrations of 60 nM and 100 nM, respectively roughly representing the 40‐fold *K*_i_ value derived from the binding experiment. As a reference β‐FNA was used at 1 μM. Preincubation was run for 60 min. Generally incubation was stopped by centrifugation and the amount of reversibly bound ligand was washed out for four times (resuspension of the memebranes in buffer for 30 min followed by centrifugation). Washed membranes were used for radioligand binding experiments with [^3^H]diprenorphine to determine the remaining specific binding at the receptor according to the standard protocols for radioligand binding. Non‐specific binding was determined in the presence of 10 μM naloxone. Data analysis was performed by normalizing the receptor bound radioactivity derived from unspecific binding equal to 0 % and total binding equal to 100 %.

Average values from three individual experiments determined in quatruplicates show no blocking of the radioligand binding site of the receptor by **9 b** or **16 b**.

**Accumulation of inositol mono phosphate (IP‐One Assay)**. Determination of the activation of μ‐OR was measured applying the IP‐One HTRF^®^ assay (Cisbio, Codolet, France) according to the manufacturer's protocol and as described previously.^53^ In brief, HEK‐293T cells were grown to confluence of approximately 70 % and transiently transfected with the cDNA of the human μ‐OR receptor applying the TransIT‐293 Mirus transfection reagent (Peqlab, Erlangen, Germany). After one day cells were detached from the culture dish with Versene (Life Technologies GmbH, Darmstadt, Germany), seeded into black 384‐well plates (10000 cells/well) (Greiner Bio‐One, Frickenhausen, Germany) and maintained for 24 h at 37 °C. After incubation with the test compounds dissolved in stimulation buffer (final range of concentration from 1 pM up to 10 μM) for 180 min at 37 °C the detection reagents were added (IP1‐d2 conjugate and Anti‐IP1cryptate TB conjugate each dissolved in lysis buffer) and incubation was continued for further 1 h at room temperature. Time resolved fluorescence resonance energy transfer (HTRF) was determined using the Clariostar plate reader (BMG, Ortenberg, Germany). In the agonist mode each compound was tested in duplicates in 5–6 individual experiments in comparison to the reference compound DAMGO (*n*=10). Antagonist properties were determined after preincubation of the test compound for 30 min, subsequent addition of a fixed concentration of the reference agonist carbachol at a final concentration of 30 nM and continued incubation for 90 min at 37 °C (5 experiments each). The resulting dose response curves were analyzed by nonlinear regression using the algorithms in PRISM 6.0 (GraphPad software, San Diego, CA), fitted with a sigmoid model and normalized to basal activity (0 %) and the maximal effect caused by the reference full agonist DAMGO (100 %) (agonist mode).

**Recruitment of β‐arrestin‐2 (PathHunter Assay)**. Measurement of arrestin‐2 recruitment was done applying the PathHunter^®^ assay (DiscoverX, Birmingham, U.K.) according to the manufacturer's protocol and as described previously.^53^, ^57^ In brief, HEK‐293 cells stably expressing the enzyme acceptor (EA)‐tagged β‐arrestin‐2 fusion protein were transiently co‐transfected with the ProLink‐tagged μ‐OR‐PK1 and GRK2 (1 : 1) employing the Mirus TransIT‐293 transfection reagent. After 24 h, cells were transferred into white clear bottom 384‐well plates (5000 cells/well) (Greiner Bio‐One) and maintained for further 24 h at 37 °C, 5 % CO_2_. To start receptor stimulated arrestin recruitment test compounds were added to the cells to get a final concentration in a range of 10 pM to 100 μM. Incubation was continued for 90 min at 37 °C. Stimulation was stopped by addition of the detection mix and incubation was continued for 60 min at room temperature. Chemiluminescence was determined using a Clariostar plate reader. Agonist mode was measured in duplicates (3 individual experiments for each compound in comparison to DAMGO (*n*=9). Antagonist properties were determined after preincubation of the test compound for 30 min, subsequent addition of 1 μM of DAMGO and continued incubation for 90 min at 37 °C (4 experiments each). Data analysis was done as described above.

**Test on cytotoxicity**. Determination of cytotoxic effects was measured with untransfected HEK293T cells in presence of 100 nM of the test ligands **9 b**, **16 b** and the reference naloxone (65‐fold *K*_i_, 30‐fold *K*_i_ and 25‐fold *K*_i_, respectively). For this, cells were seeded in a 12‐well plate (300,000 per well) and kept growing for 24 h in full medium (DMEM/F12 supplemented with 10 % FBS, penicillin‐streptomycin and L‐glutamine; Life Technologies, Darmstadt, Germany). Test compounds were diluted in PBS with an equal amount of DMSO and added to the cells at a final concentration of 100 nM followed by a continued incubation for further 24 h. Finally, cells were thoroughly observed in a microscope for any change in morphology and growing behavior different to vehicle or reference. After that cells were detached (Versene, Life Technologies) and the cell number of each well was determined in an automated cell counter (Countess, Life Technologies). The number of cells from six individual experiments was normalized relative to vehicle (100 %) subsequently calculating an average mean value±SEM.

**Microsomal stability assay**. The metabolism studies were performed with pooled microsomes from male rat liver (Sprague‐Dawley) which were used as purchased from Sigma Aldrich and were stored at −75 °C until usage. Nicotinamide adenine dinucleotide phosphate (NADPH) from Sigma Aldrich was stored at −8 °C and also used as purchased. The microsomal degradation experiments were performed in polyethylene tubes from Eppendorf with 1.5 mL volume size. The incubation mixture with a total volume of 1 mL contained the azocarboxamides, cinnamides or imipramine as test substances (40 μM stock, pre‐diluted from a 0.1 mM stock in DMSO), microsomes in a concentration of 1 mg of microsomal protein/mL of incubation mixture and Tris‐MgCl_2_ buffer (48 mM Tris, 4.8 mM MgCl_2_ **⋅**6 H_2_O, pH 7.4). The microsomal reactions were initiated by the addition of a NADPH solution (100 μL, final concentration of 1.1 μM) and performed at 37 °C. After time intervals of 0, 20, 40 and 60 minutes, samples (100 μL) were drawn from which the enzymatic reactions were terminated by addition of ice‐cold acetonitrile (200 μL, containing the internal standard **22**), and precipitated protein was removed by centrifugation (15000 rcf for 3 min) and the solids removed by filtration. The supernatant was analyzed by LC/MS (flow rate 0.5 mL/min, binary solvent system of 0.1 % formic acid in water and 0.1 % formic acid in acetonitrile, gradient: 20 %→90 % 0.1 % formic acid over 30 min). Parallel control incubations were conducted in the absence of enzyme cofactor NADPH as negative controls and for the determination of unspecific binding to matrix. The concentrations of the remaining substrates were calculated as a mean value±SEM. of three independent experiments by comparison of the area under the curve (AUC) of the remaining substrate in comparison to the AUC of substrate at time 0. Estimating a similar ionization rate, internal fluctuations were corrected by a factor calculated from the AUC of the internal standard at each time point.

**Radiosynthesis**. No‐carrier‐added [^18^F]fluoride in target water was provided by Universitätsklinikum Würzburg, Klinik und Poliklinik für Nuklearmedizin, Germany. Thin layer chromatography (TLC) was carried out on silica gel‐coated plastic sheets (Polygram®, Sil G/UV_254_, Macherey Nagel). Electronic autoradiography (Instant Imager TM, Canberra Packard) was used to analyze radio‐TLC data. The HPLC system (Series 1100, Agilent) was equipped with a VWD lamp (detection at 254 nm) and additionally connected to a radio‐detector (500 TR Series, Packard).

[^18^F]**11b** was prepared as described previously.^41^ After completion of the reaction, the yellow solution containing [^18^F]**11b** was diluted with HCl (0.2 m, 15 mL), passed through a cartridge (SepPak tC18, Waters) and the cartridge was washed with CH_3_CN /0.2 M HCl (20 : 80, 5 mL) and H_2_O (2 mL). [^18^F]**11b** was eluted with 1 mL of ethanol in a reaction vial which was prepared with the required reactants (the primary amine **10** (54 μmol or 108 μmol) and Cs_2_CO_3_ (7.5 mg, 23 μmol)). The reaction mixture was stirred at room temperature and the radiochemical yield was determined from aliquots taken from the reaction mixture after 2, 5 and 10 min by radio‐TLC (ethanol/CH_2_Cl_2_ 9 : 1, *R*_f_ ([^18^F]**9b**)=0.4). After 10 min, [^18^F]**9b** was isolated by semi‐preparative radio‐HPLC (Kromasil C8, 125×8 mm, 4 mL/min, solvent: A: water (0.1 % TFA), solvent B: acetonitrile (0.1 % TFA), gradient A/B: 75 : 25 to 40 : 60 in 30 min, *t*_R_=11.4 min) and coinjected together with reference standard **9 b**.

## Conflict of interest

The authors declare no conflict of interest.

## Supporting information

As a service to our authors and readers, this journal provides supporting information supplied by the authors. Such materials are peer reviewed and may be re‐organized for online delivery, but are not copy‐edited or typeset. Technical support issues arising from supporting information (other than missing files) should be addressed to the authors.

Supplementary
