# Supplementary material for: Synthesis, Radiosynthesis and Biological Evaluation of Buprenorphine‐Derived Phenylazocarboxamides as Novel μ‐Opioid Receptor Ligands
Source: ChemMedChem. 2020 Jun 2;15(13):1175–86. doi: 10.1002/cmdc.202000180 (PMC7383964; doi:10.1002/cmdc.202000180)
Supplement: Supplementary file 1 — Supplementary [file CMDC-15-1175-s001.pdf]

ChemMedChem

Supporting Information

Synthesis, Radiosynthesis and Biological Evaluation of Buprenorphine-Derived Phenylazocarboxamides as Novel μ-Opioid Receptor Ligands

Jasmin Krüll, Stefanie K. Fehler, Laura Hofmann, Natascha Nebel, Simone Maschauer, Olaf Prante, Peter Gmeiner, Harald Lanig, Harald Hübner, and Markus R. Heinrich*© 2020 The Authors. Published by Wiley-VCH Verlag GmbH & Co. KGaA.
This is an open access article under the terms of the Creative Commons Attribution License, which permits use, distribution and reproduction in any medium, provided the original work is properly cited.

Table of content

Overview of synthesized intermediates and ligands S3

Docking S4

Receptor Model Generation S4

Docking Studies S4

Determinations of half-life ($t_{1/2}$) and intrinsic clearance (CL'_{int}) S7

Radiosynthesis of [^{18}F]9b S7

Purity Determination S8

References S9

Overview of synthesized intermediates and ligands

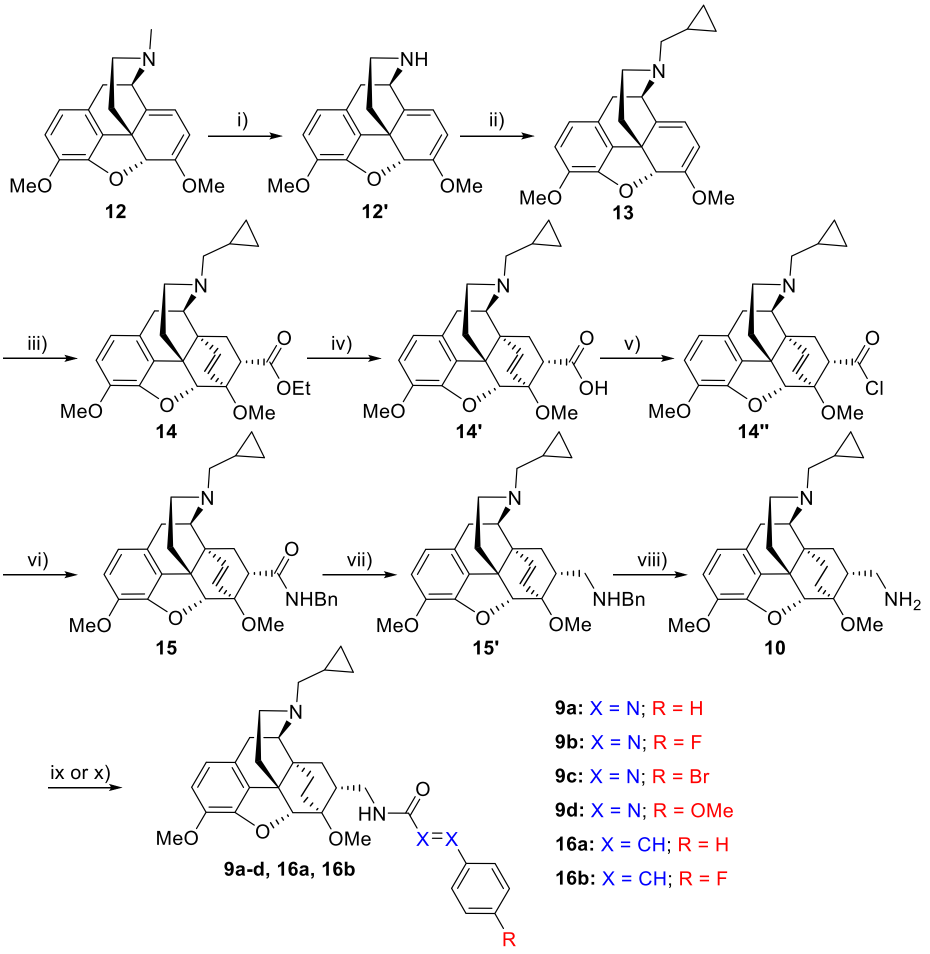

Scheme S1: Overview of synthesized intermediates and ligands.

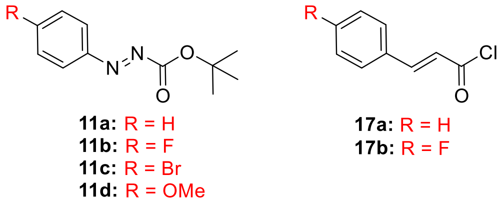

Figure S1: Overview of used azocarboxylic esters and cinnamic acid chlorides.

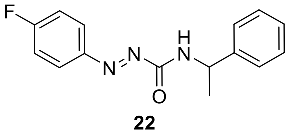

Figure S2: Internal standard used for metabolism assay.

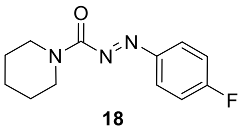

Figure S3: Further carboxamide for comparison of microsomal stability.

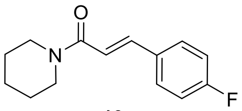

19

Figure S4: Further cinnamide for comparison of microsomal stability.

Docking

Receptor Model Generation

The opioid receptor geometries were modelled applying the SWISS-MODEL web service (accessible via http://swissmodel.expasy.org) using available X-ray structures (PDB database entries μ-OR: 4DKL.pdb; δ -OR: 4N6H.pdb; κ-OR: 4DJH.pdb) as templates. Co-crystallization with antagonist ligands ensures that all receptor geometries represent the inactive state and are therefore directly comparable by geometric superposition. Application of the modelling procedure on the X-ray structures ensures that eventually missing sidechain atoms in the template structure are added, additional proteins like lysozyme or cytochrome removed, and obtaining the correct amino acid sequence (UniProt database entries μ-OR: P35372; δ -OR: P41143; κ-OR: P41145) for the receptors.

Docking Studies

The three-dimensional structures of all ligands under investigation were built and geometry-optimized using Avogadro (available via https://avogadro.cc). The nitrogen atom of the morphine scaffold was treated explicitly protonated. All docking studies were performed using AutoDock VINA (available via http://vina.scripps.edu) applying an established standard docking protocol.[1] For the preparation of the protein and ligand input files, the AutoDockTools (available via http://mgltools.scripps.edu) were used. Ligands were treated fully flexible (including free translation and rotation of the whole molecule), the protein sidechains were considered rigid. All docking runs were repeated with different levels of exhaustiveness. For every ligand, the best docking pose was selected according to VINA's built-in free energy-based scoring function.

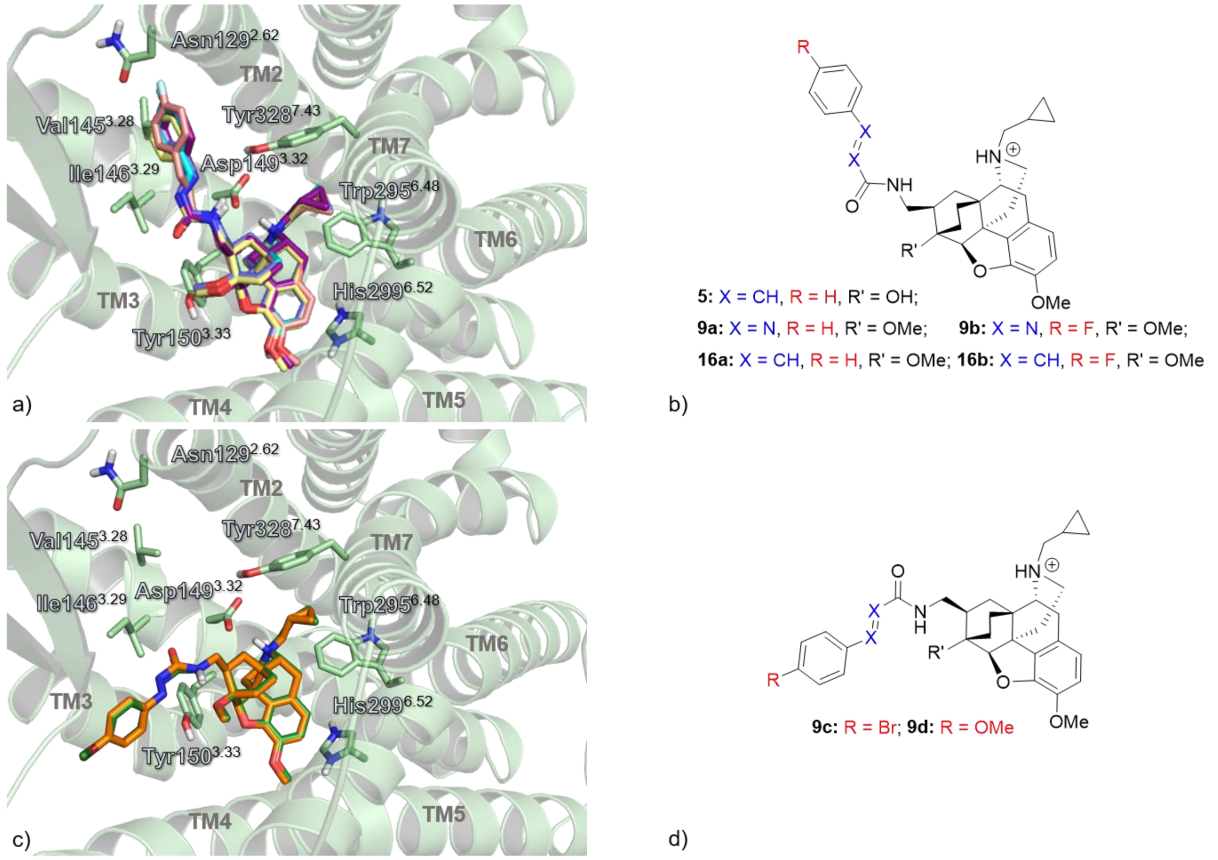

Figure S5. Docking into the μ-OR subtype (derived from PDB: 4DKL). **a)** Best ranked poses of OR ligands **5**, **9a**, **9b**, **16a** and **16b** in the μ-OR subtype (binding mode 1). **b)** Structures of ligands **5**, **9a**, **9b**, **16a** and **16b**. **c)** Best ranked poses of OR ligands **9c** and **9d** in the μ-OR subtype (binding mode 2). **d)** Structures of ligands **9c** and **9d**. The structures were visualized using PyMOL 1.3 and illustrated in the following color code: **5** (blue), **9a** (yellow), **9b** (purple), **9c** (orange), **9d** (green), **16a** (cyan), **16b** (salmon).

Within the μ-OR subtype, the morphinan scaffold of the ligands **5**, **9a-9d**, **16a** and **16b** adopt the same orientation, whereas the side chain flips dependent on the attached substituents (Figure S5). All ligands with no or relatively small substituents in 4-position of the phenylazocarboxamide (**5**, **9a**, **9b**, **16a** and **16b**) present the same binding mode (binding mode 1, Figure S5a). In contrast, larger substituents such as 4-methoxy or 4-bromo on the azocarboxamides **9c** and **9d** lead to a different binding mode (binding mode 2, Figure S5c) in the μ-OR subtype, which however results in similar binding affinities (see Table 2).

The preferred binding modes found for the ligands **5**, **9a-9d**, **16a** and **16b** in the κ-OR do not show a significant dependence on the structural variations so that there is an almost full overlap of all seven structures (Figure S6). In comparison to the binding modes predicted for the μ-OR subtype (Figure S5), the docking studies at the κ-OR however suggest a completely different position of the morphinan scaffold in the binding pocket. This structural re-orientation could be due to the fact the κ-OR binding pocket is narrower than those of the μ-OR and δ-OR, accompanied by a sterically more demanding side chain decoration of the backbone. In seeming contradiction to this finding, the structures for all three OR subtypes used as templates correspond to inactive, antagonist-bound states, as reference compound **5** and the novel ligands

9b and **16b** have been determined to be antagonists. Nevertheless, the almost identical binding modes determined for **9a-d** and **16a,b** correspond well to the similar binding affinities of the compounds to the κ-OR subtype (10-25 nM, Table 2).

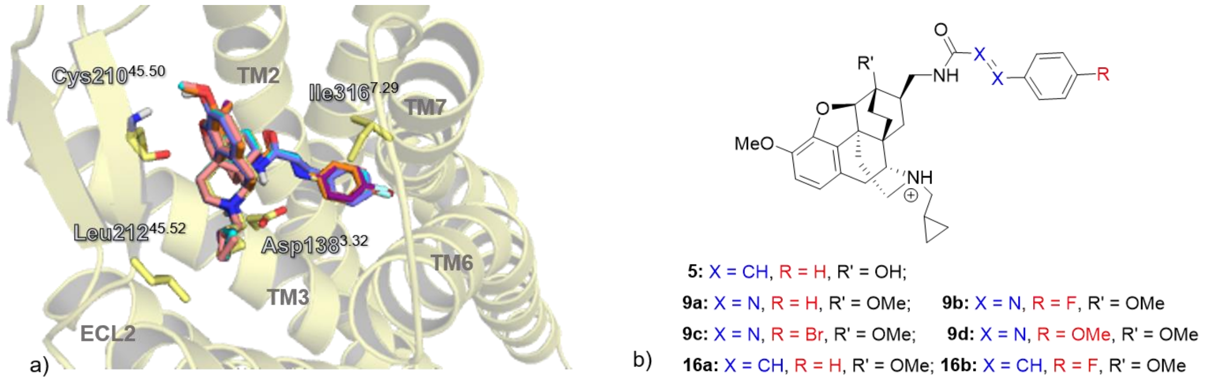

Figure S6. Docking into the κ-OR subtype (derived from PDB: 4DJH). **a)** Best ranked poses of OR ligands **5**, **9a-9d**, **16a** and **16b** in the κ -OR subtype. **b)** Structures of ligands **5**, **9a-9d**, **16a** and **16b**. The structures were visualized using PyMOL 1.3 and illustrated in the following color code: **5** (blue), **9a** (yellow), **9b** (purple), **9c** (orange), **9d** (green), **16a** (cyan), **16b** (salmon).

All ligands **5, 9a-d** and **16a,b** are also likely to present very similar binding modes within the δ-OR subtype (Figure S7), whereat the overall orientation of the ligands is comparable to binding mode 2, which was only observed for the two bulkier ligands **9c** and **9d** in the µ-OR subtype (Figure S7c). While the azocarboxamide side chain shows slight deviations in orientation, the morphinan scaffold of the seven ligands adopts exactly the same position in the δ-OR, which is again in agreement with the narrow range of binding affinities (28-63 nM, Table 2).

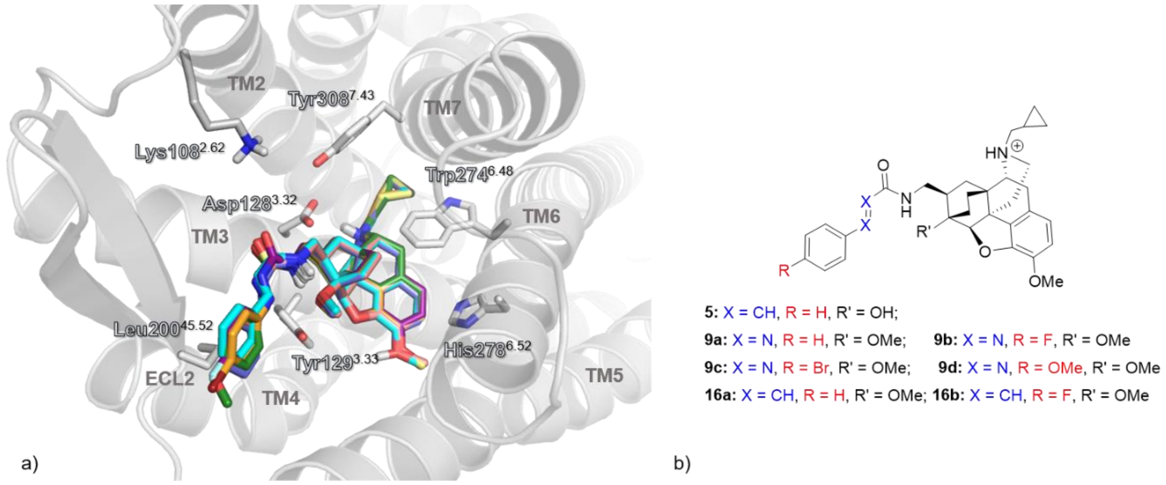

Figure S7. Docking into the δ-OR subtype (derived from PDB: 4N6H). **a)** Best ranked poses of OR ligands **5**, **9a-9d**, **16a** and **16b** in the δ-OR subtype. **b)** Structures of ligands **5**, **9a-9d**, **16a** and **16b**. The structures were visualized using PyMOL 1.3 and illustrated in the following color code: **5** (blue), **9a** (yellow), **9b** (purple), **9c** (orange), **9d** (green), **16a** (cyan), **16b** (salmon).

Determinations of half-life ($t_{1/2}$) and intrinsic clearance (CL'_{int})

The concentrations of the remaining substrates were plotted in their respective logarithmic form as a function of the time. With aid of the regression line (gradient) of the in this way received function, the elimination rate constant (k) was determined which was furthermore used for the calculation of the half-life ($t_{1/2}$) and intrinsic clearance ($CL'int$) according to the following equations published by *Obach et al*:[2]

(I) k = -gradient

(II) $t_{1/2}\,[\text{min}] = \frac{0.693}{k}$

(III) $$CL'_{in} = \left[\frac{\mu L}{\min \times mg\ (microsome)}\right] = \frac{0.693}{t_{1/2}\,[min]} \times \frac{total\ incubation\ volume\ [\mu L]}{microsome\ [mg]}$$

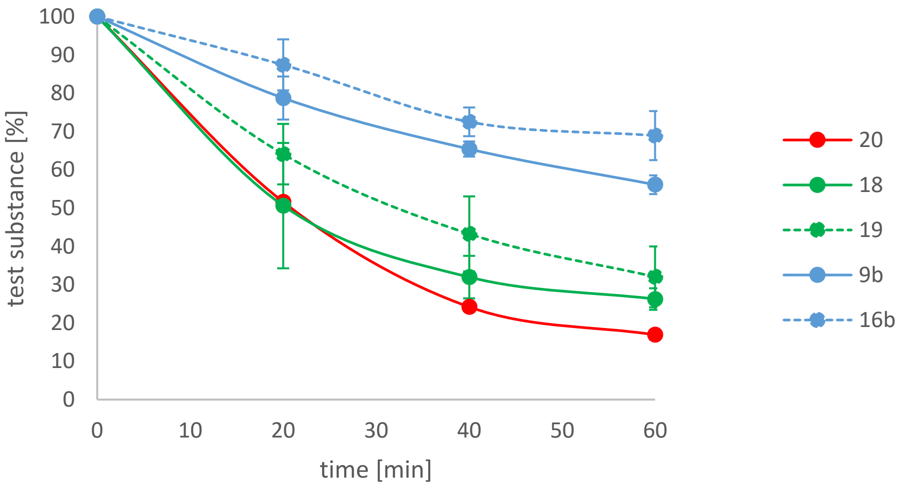

Figure S8: Metabolic stability of the phenylazocarboxamides **18** and **9b** and the cinammides **19** and **16b** as well as imipramine **20** as positive control.

Radiosynthesis of [^{18}F]9b

[^{18}F]11b was prepared as described previously. After completion of the reaction, the yellow solution containing **[^{18}F]11b** was diluted with HCl (0.2 M, 15 mL), passed through a cartridge (SepPak tC18, Waters) and the cartridge was washed with CH_3CN /0.2 M HCl (20:80, 5 mL) and H_2O (2 mL). **[^{18}F]11b** was eluted with 1 mL of ethanol in a reaction vial which was prepared with the required reactants (the primary amine **10** (54 µmol or 108 µmol) and Cs_2CO_3 (7.5 mg, 23 µmol)). The reaction was stirred at room temperature and the radiochemical yield was determined from aliquots taken from the reaction mixture after 2, 5 and 10 min by radio-TLC (ethanol/dichloromethane = 9:1, R_f (**[^{18}F]9b**) = 0.4). After 10 min **[^{18}F]9b** was isolated by semi-preparative radio-HPLC (Kromasil C8, 125 x 8 mm, 4 mL/min, solvent: A: water (0.1 % TFA), solvent B: acetonitrile (0.1 % TFA), gradient A/B: 75:25 to 40:60 in 30 min, t_R=11.4 min) and coinjected together with reference standard **9b** (see Fig. S3).

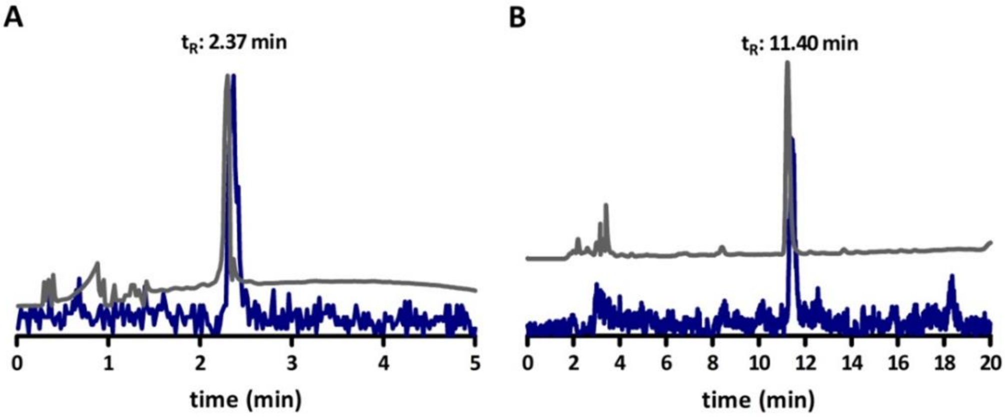

Figure S9. Coinjection of **[^18F]9b** (blue line) with its reference compound **9b** (grey line) using two different HPLC methods. A) Chromolith RP-18e, 100 x 4.6 mm, flow rate: 4 mL/min, solvent A: water (0.1 % TFA), solvent B: acetonitrile (0.1 % TFA), gradient A/B: 90:10 to 50:50 in 5 min. B). Kromasil C8, 125 x 8 mm, flow rate: 4 mL/min, solvent: A: water (0.1 % TFA), solvent B: acetonitrile (0.1 % TFA), gradient A/B: 75:25 to 40:60 in 30 min.

Purity Determination

The purity of the compounds was determined *via* HPLC analysis using the area under the curve (AUV) of the compound signal compared to further observed signals. The HPLC analysis was performed using the gradient: 5:95→100:0 ACN/H_2O+0.1% TFA over 20 min and UV signal of the wavelength λ = 254 nm

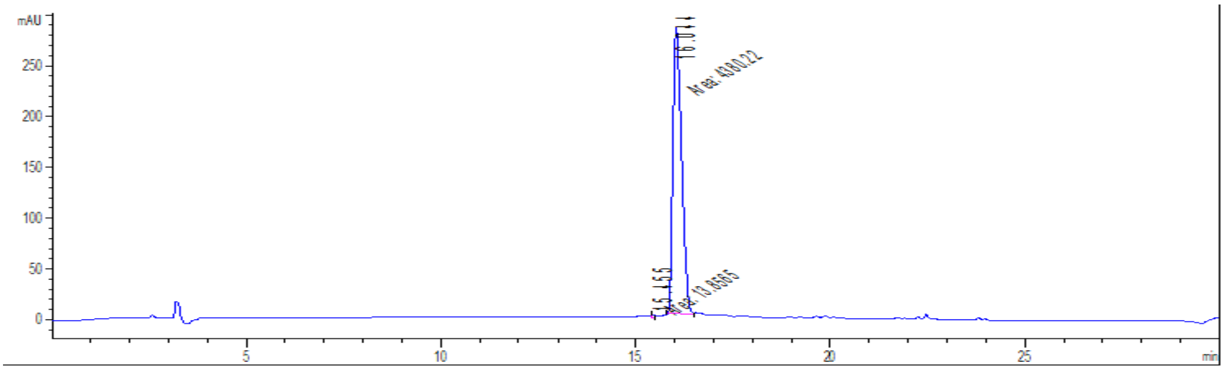

Figure S10: Chromatogram of azocarboxamide **9a**.

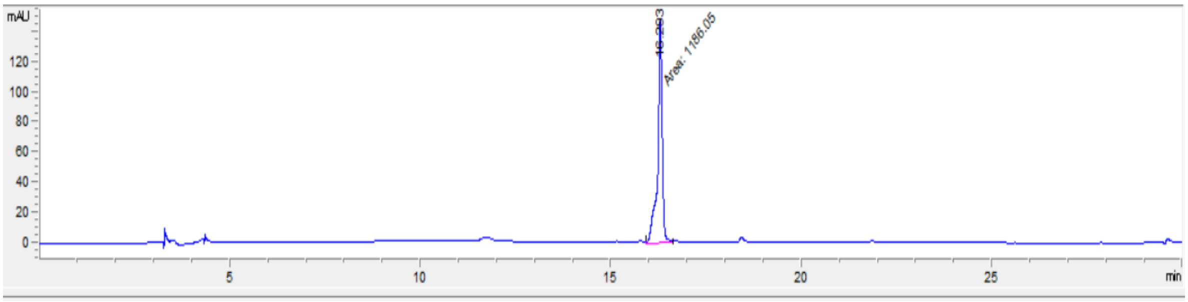

Figure S11: Chromatogram of azocarboxamide **9b**.

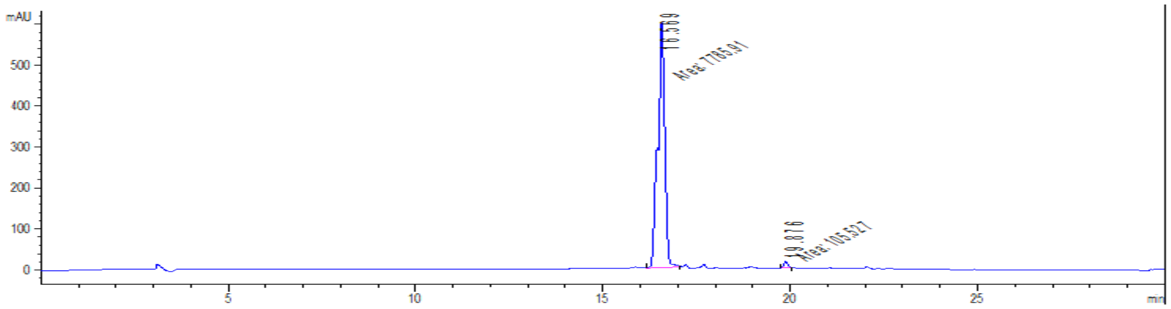

Figure S12: Chromatogram of cinnamide **16a**.

References

[1] O. Trott, A. J. Olson, *J. Comput. Chem.* **2010**, *31*, 455-461.
[2] R. S. Obach, *Drug Metab. Dispos.* **1999**, *27*, 1350-1359.
[3] A. Pfeiffer, A. Pasi, P. Mehraein, A. Herz, *Brain Res*. **1982**, *248*, 87–96.
[4] R. F. Dannals, H. T. Ravert, J. J. Frost, A. A. Wilson, H. D. Burns, H. N. Wagner, *Int. J. Appl. Radiat. Isot.* **1985**, *36*, 303-306
[5] J. Marton, B. W. Schoultz, T. Hjørnevik, A. Drzezga, B. H. Yousefi, H.-J. Wester, F. Willoch, G. Henriksen, *J Med Chem.* **2009**, *52*, 5586–5589.
[6] H. J. Wester, F. Willoch, T. R. Tölle, F. Munz, M. Herz, I. Oye, J. Schadrack, M. Schwaiger, P. Bartenstein, *J. Nucl. Med*. **2000**, *41*, 1279-1286.
[7] R. J. Riss, Y. T. Hong, J. Marton, D. Caprioli, D. J. Williamson, V. Ferrari, N. Saigal, B. L. Roth, G. Henriksen, T. D. Fryer, J. W. Dalley, F. I. Aigbirhio, *J. Nucl. Med.* **2013**, *54*, 299-305.
